# Sustainable Recycled Aggregate Concrete Incorporating Volcanic Ash and Basalt Fibers with Enhanced Mechanical, Durability, and Environmental Performance

**DOI:** 10.1021/acsomega.6c04151

**Published:** 2026-08-07

**Authors:** Zehra Funda Akbulut, Soner Guler

**Affiliations:** † Department of Mining Engineering, Faculty of Engineering, 53000 University of Van Yüzüncü Yıl, Van 65080, Turkey; ‡ Department of Civil Engineering, Faculty of Engineering, University of Van Yüzüncü Yıl, Van 65080, Turkey

## Abstract

This study evaluates the mechanical, durability, statistical, and environmental performance of recycled aggregate concrete (RAC) incorporating volcanic ash (VA) as a partial cement replacement and basalt (BA) fibers as reinforcement. VA was used at 5%, 10%, and 15%, followed by BA fiber additions of 0.25%, 0.50%, and 1% by volume. Fresh density, water absorption (WA), rapid chloride permeability (RCPT), compressive strength (CS), flexural strength (FS), and life-cycle environmental impacts were systematically assessed, and regression analyses were conducted to quantify relationships among parameters. Replacing cement with 10% VA reduced WA from 8.78% to 8.21% (6.49% reduction) and RCPT from 3917 to 3712 coulombs (5.23% reduction), indicating improved pore refinement and chloride resistance. A strong correlation between WA and RCPT (R^2^ = 0.849) confirmed that ion permeability is primarily governed by capillary porosity. Moderate negative correlations were observed between durability parameters and CS, with R^2^ values of 0.592 (WA–CS) and 0.682 (RCPT–CS). The highest CS (38.64 MPa) was achieved with 10% VA and 0.25% BA fibers, representing a 3.79% increase over the control. Increasing fiber content to 1% reduced CS by 10.98%. FS exhibited only minor variations, with a maximum increase of 2.55% compared to the control mixture, but showed stronger correlations with durability indicators (R^2^ = 0.776 for WA–FS and R^2^ = 0.676 for RCPT–FS) than with CS (R^2^ = 0.592). Life cycle assessment (LCA) revealed a 10.46% reduction in global warming potential (GWP) at 10% VA. Overall, 10% VA with 0.25% BA fibers provides the most balanced performance.

## Graphical abstract

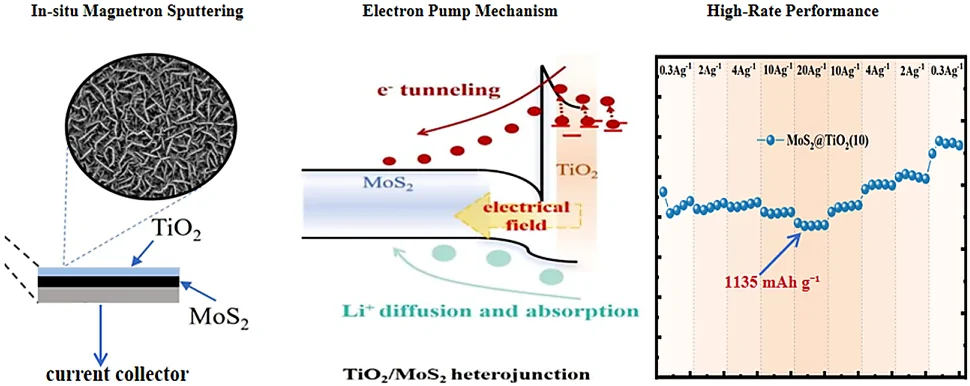

## 1 Introduction

The construction sector is one of the largest consumers of natural resources and a major contributor to global carbon emissions, primarily due to the intensive use of Portland Cement (PC) and natural aggregates. Increasing environmental concerns have therefore intensified research into the reuse of construction and demolition waste (CDW) and the replacement of PC with supplementary cementitious materials (SCMs).^1^ Among the proposed strategies, RAC is particularly promising because it simultaneously reduces landfill disposal and preserves natural aggregate reserves.^2^

Despite its sustainability advantages, RAC generally exhibits higher porosity, weaker interfacial transition zones (ITZs), and lower durability than conventional concrete.^3–5^ These deficiencies mainly originate from the old mortar adhered to recycled aggregates and the heterogeneous surface characteristics of these aggregates.^6^ The porous residual mortar increases water absorption and facilitates the ingress of aggressive ions, such as chlorides and sulfates, thereby adversely affecting long-term durability and mechanical performance.^7^ Previous studies have shown that RAC frequently exhibits inferior construction-related and structural performance compared with natural aggregate concrete (NAC), particularly when recycled aggregate replacement ratios exceed 50%, and especially under full replacement (100%). The literature further indicates that fully recycled aggregate systems may exhibit increased porosity, higher water absorption, reduced durability, and weaker bond strength due to the heterogeneous, porous nature of recycled aggregates.^8^

Ramalingam et al.^9^ reported that untreated RAC exhibited approximately 28.59% lower compressive strength and 47.51% higher water absorption than NAC due to the weak residual mortar attached to recycled aggregates. The same study demonstrated that interfacial and matrix modification strategies, including carbonation and acid treatment, significantly improved RAC performance by reducing pore connectivity and strengthening the aggregate–paste interface. Carbonation treatment, for example, increased compressive strength by approximately 16.44% compared with untreated RAC.^9^ These findings highlight the importance of matrix densification and interfacial enhancement strategies for developing durable and structurally reliable recycled aggregate concrete systems.

The incorporation of SCMs is one of the most effective approaches for improving the microstructure of RAC. Materials such as fly ash, silica fume, and ground granulated blast furnace slag have been extensively investigated for this purpose.^10^ These materials reduce porosity through filler action and secondary hydration reactions, resulting in a denser ITZ.^11^ However, their availability often depends on industrial byproducts and regional production capacity, which may limit their long-term sustainability and accessibility.^12^ Consequently, naturally occurring pozzolans have attracted increasing attention as alternative SCMs. Among these materials, VA is particularly attractive because of its widespread availability, low processing requirements, and relatively low environmental impact.^13^ The pozzolanic reaction between VA and calcium hydroxide promotes the formation of additional calcium–silicate–hydrate (C–S–H) gel, leading to pore refinement and improved durability characteristics.^14^

Nevertheless, the use of VA introduces new challenges. Its angular and highly porous particles increase internal friction, thereby reducing workability and increasing water demand.^15^ This problem becomes more pronounced in RAC mixtures because recycled aggregates already absorb a considerable amount of mixing water.^16^ Consequently, the combined use of VA and recycled aggregates may produce mixtures with insufficient compaction and heterogeneous microstructure unless carefully optimized.^17^ Previous studies have reported both improvements and reductions in strength depending on VA replacement level, indicating that the balance between pozzolanic densification and workability loss governs performance.^18^

Fibers are commonly incorporated to compensate for the brittleness of cementitious composites. Steel, polypropylene, and poly­(vinyl alcohol) fibers have been extensively studied in both conventional concrete and RAC.^19^ Fibers primarily improve crack control and postcracking behavior rather than peak compressive strength, and their effectiveness strongly depends on dispersion quality and bond characteristics at the fiber–matrix interface.^20^ Recently, BA fibers have attracted increasing interest as an environmentally favorable reinforcement material because of their high tensile strength, thermal stability, and chemical resistance, and because they require less energy to produce than synthetic fibers.^21^

However, the influence of BA fibers on mechanical strength remains inconsistent in the literature. While some studies report improvements in tensile or flexural behavior, others observe negligible or even negative effects on compressive strength due to fiber clustering and entrapped air.^22^ This effect becomes critical in RAC, where weak ITZ regions already exist. Inadequate fiber dispersion may further deteriorate matrix continuity, whereas a well-bonded interface may instead bridge microcracks and delay crack propagation.^23^ Therefore, the performance of fiber-reinforced RAC depends on the interaction between matrix densification and fiber distribution rather than on fiber addition alone.^24^

From a sustainability perspective, the environmental performance of modified concrete mixtures must be evaluated using LCA.^25^ Replacing cement with SCMs typically reduces global warming potential, while recycled aggregates decrease resource depletion.^26^ Fiber addition, however, may partially offset these benefits, depending on production energy intensity and dosage.^27^ Therefore, a comprehensive assessment should simultaneously consider mechanical, durability, and environmental performance.

Although RAC, VA, and BA fibers have been widely investigated individually, their combined performance in fully recycled-aggregate concrete systems remains poorly understood.^28^ In particular, the interactive mechanisms governing matrix densification, fiber–matrix bonding, durability, and environmental sustainability under 100% recycled-aggregate replacement conditions have not been systematically clarified.^29^ Moreover, limited studies have simultaneously evaluated mechanical properties, durability indicators, microstructural characteristics, and environmental impacts within a unified experimental framework.

Accordingly, the present study investigates the combined effects of volcanic ash and basalt fibers on the mechanical, durability, microstructural, statistical, and environmental performance of fully recycled aggregate concrete. The novelty of the study lies in integrating compressive strength, water absorption, rapid chloride permeability, SEM-based microstructural characterization, regression-based correlation analysis, and cradle-to-gate life cycle assessment within a single experimental framework. Furthermore, the study evaluates whether VA-induced matrix refinement can improve fiber–matrix interfacial efficiency and mitigate the inherently weak ITZ associated with recycled aggregates. By linking microstructural observations to durability, mechanical, and environmental performance indicators, this research provides a comprehensive assessment and optimization strategy for sustainable fully recycled-aggregate concrete mixtures.

## 2 Experimental Program

### 2.1 Materials

Concrete mixes were produced using PC, VA, BA fibers, recycled aggregates, tap water, and a polycarboxylate ether-based superplasticizer (SP) (Sika ViscoCrete-1050 SA, Sika, Türkiye). The SP was used to improve workability and maintain mixture homogeneity throughout the mixing process. The SP dosage was adjusted based on the VA replacement ratio and BA fiber content to maintain adequate workability and mixture stability. The control mixture (K0) contained 0.15% SP by weight of binder. With increasing VA replacement levels, the SP dosage was gradually increased to 0.19%, 0.23%, and 0.25% for K1, K2, and K3, respectively, to compensate for the increased fineness and water demand of VA. In the fiber-reinforced mixtures, higher SP dosages were required due to the reduced workability caused by the incorporation of BA fibers. Accordingly, SP dosages of 0.36%, 0.54%, and 1.00% were used for K4, K5, and K6, respectively. The PC used in this study was a CEM I 42.5 R type supplied by the Mersin Çimsa Factory (Türkiye). Municipal tap water was used for both mixing and curing.

The VA was obtained from natural volcanic deposits in the Patnos district of Ağrı Province (Türkiye). Prior to use, the material was oven-dried at 105 °C for 24 h to eliminate residual moisture, then ground under dry conditions using a laboratory-scale ball mill to enhance its fineness and pozzolanic reactivity. The grinding process was conducted at 300 rpm for 60 min using hardened steel grinding balls with diameters of 10–20 mm. A ball-to-powder mass ratio of 5:1 was maintained throughout the milling process to ensure efficient particle-size reduction and homogeneous grinding. Following milling, the processed VA was passed through a 75 μm sieve, and only the fraction that passed was used in the experimental program. The milling end point was determined based on achieving the target fineness corresponding to the 75 μm sieve criterion. No noticeable caking or particle agglomeration was detected after grinding. Pozzolanic activity tests were performed prior to incorporating the VA into the concrete mixtures to verify its suitability as a supplementary cementitious material. Pozzolanic activity was evaluated in accordance with ASTM C618 ^30^ using the Strength Activity Index (SAI) method. Mortar specimens containing volcanic ash as a partial cement replacement were prepared and tested alongside control specimens. The SAI was calculated as the ratio of the 28-day compressive strength of the volcanic ash-containing mortar to that of the control mortar. The calculated 28-day SAI value was 79.27%, exceeding the minimum requirement of 75% specified in ASTM C618. These results confirmed the pozzolanic suitability of the volcanic ash and supported its use as a supplementary cementitious material in the present study.

BA fibers with a nominal length of 12 mm were commercially supplied and purchased through the Made-in-China online marketplace. The physical and mechanical properties provided by the supplier, including fiber diameter, density, tensile strength, elastic modulus, elongation at break, and fiber length, are summarized in Table 2. The visual appearances of the VA and the 12 mm BA fibers used in this study are presented in Figure 1.

**Figure 1:**
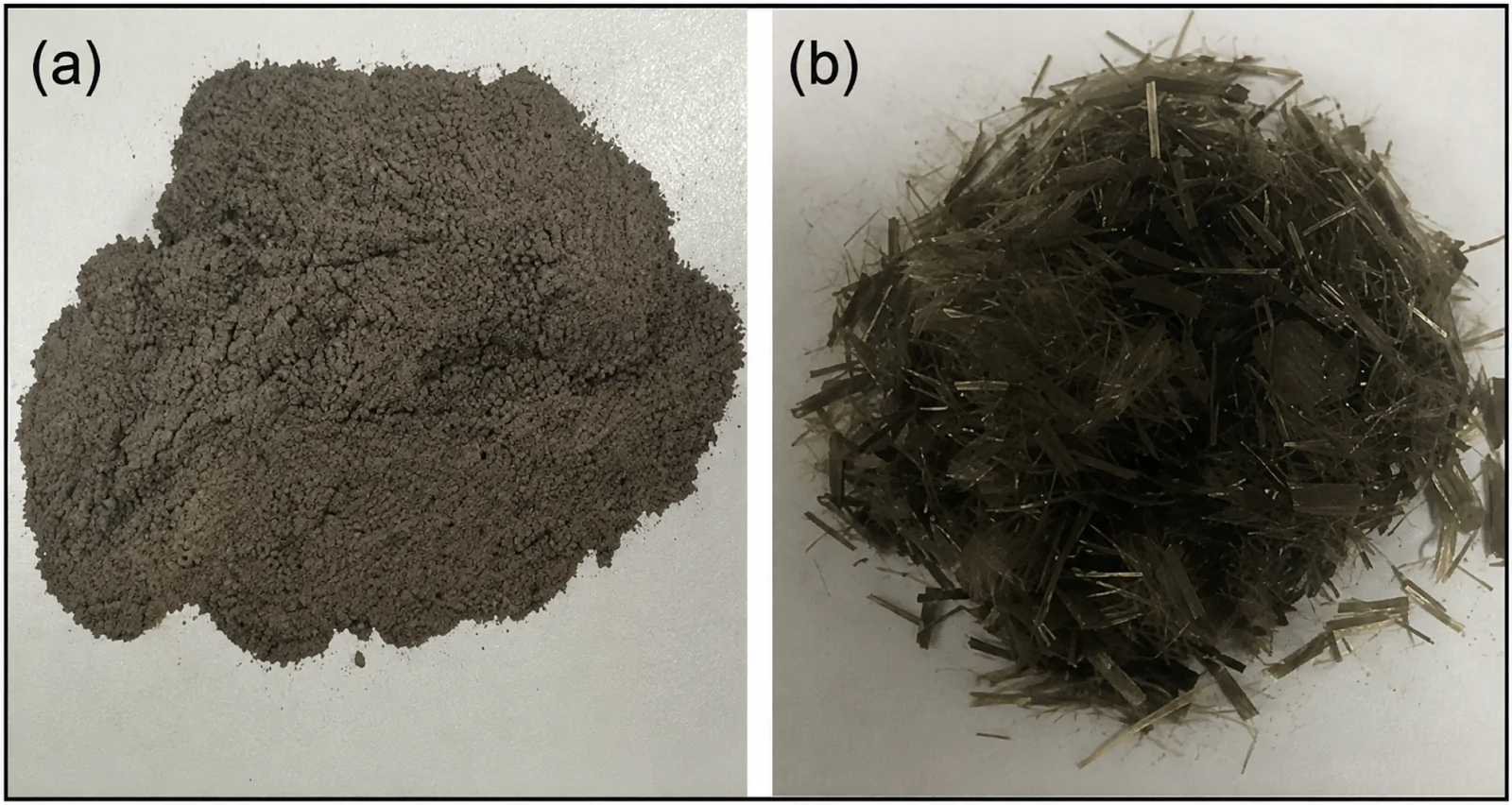
Physical appearance of the materials used in this study: (a) volcanic ash (VA) and (b) 12 mm basalt (BA) fibers.

The recycled aggregates were obtained from the demolition waste of a reinforced-concrete residential building with one basement level, a ground floor, and six upper stories, located in the İpekyolu district of Van Province (Türkiye), which was demolished as part of an urban transformation program. The debris was transported to the laboratory, crushed using a jaw crusher, and sieved to produce two aggregate fractions: 0–5 mm and 5–12 mm.

These aggregates were used in saturated-surface-dry conditions, and 100% recycled aggregates were employed in all mixtures. A general view of the demolished building, the crushing process, and the resulting recycled aggregates is shown in Figure 2. A polycarboxylate ether-based high-range water-reducing admixture was used as the SP.

**Figure 2:**
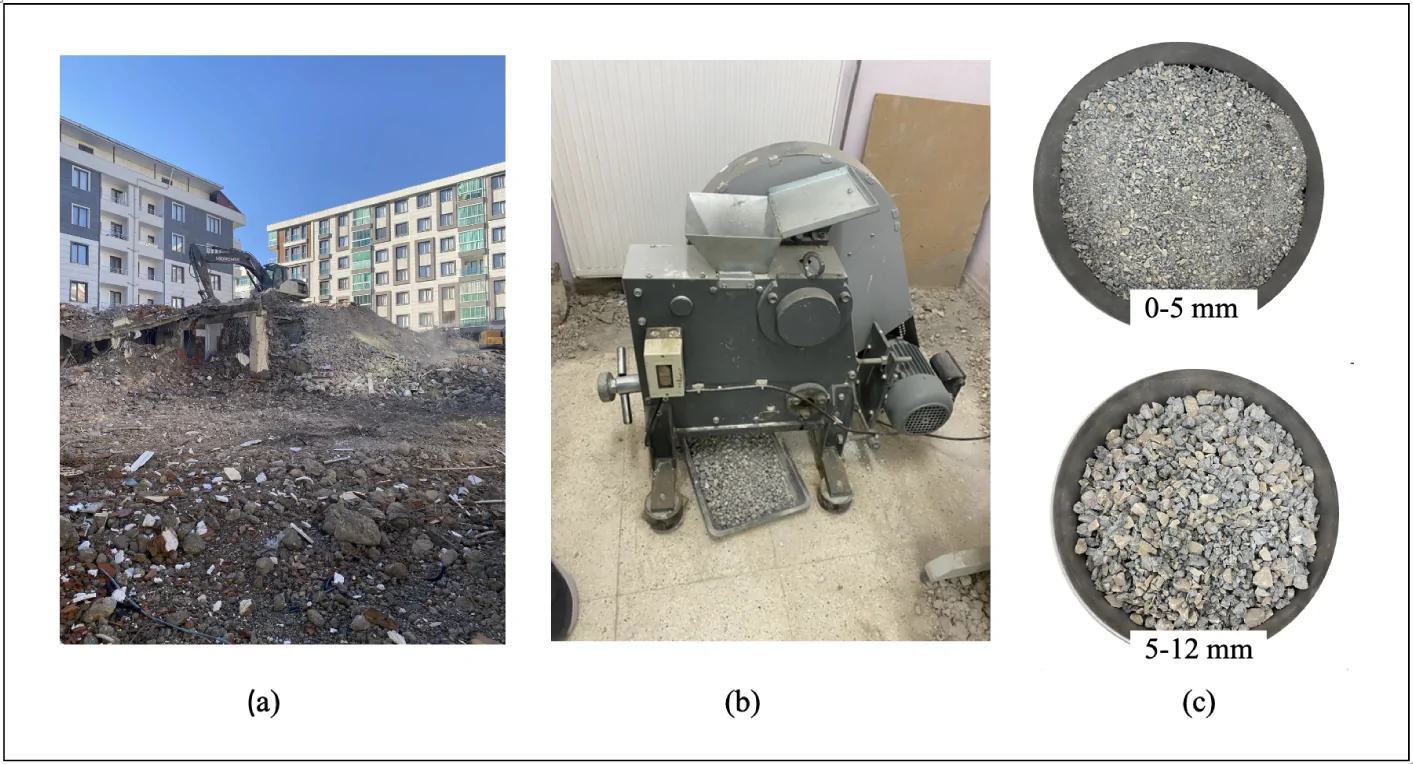
Collection, processing, and preparation of recycled aggregates: (a) demolished reinforced concrete building, (b) crushing of waste concrete in the jaw crusher, and (c) recycled aggregate fractions (0–5 mm and 5–12 mm).

The physical, chemical, and mechanical properties of PC and the main characteristics of VA are given in Table 1. The technical characteristics of the incorporated BA fibers are presented in Table 2. A typical Scanning Electron Microscopy (SEM) micrograph of VA is presented in Figure 3(a), whereas the corresponding X-ray Diffraction (XRD) pattern is illustrated in Figure 3(b).

**Table 1:** Physical, Chemical, and Mechanical Properties of CEM I 42.5 R Portland Cement (PC) and Volcanic Ash (VA) Used in This Study

Property	CEM I 42.5 R (PC)	Volcanic Ash (VA)
SiO_2_ (%)	13.41	54.60
Al_2_O_3_ (%)	3.67	14.85
Fe_2_O_3_ (%)	7.74	10.02
CaO (%)	67.43	6.50
MgO (%)	1.33	2.05
SO_3_ (%)	4.87	0.48
K_2_O (%)	0.98	2.02
Na_2_O (%)	0.38	4.12
Specific gravity	3.15	2.70
Blaine fineness (cm^2^ /g)	3357	3570
Initial setting (min)	184	–
Final setting (min)	257	–
2-day strength (MPa)	24.33	–
28-day strength (MPa)	45.59	–
Loss on ignition (%)	1.96	-

**Table 2:** Technical Specifications of the BA Fibers Used in This Study

Property	Technical specifications of BA fibers
Fiber type	Basalt (BA) fiber
Chemical origin	Volcanic basalt rock
Color	Dark brown/black
Fiber diameter (μm)	9–23
Fiber length (mm)	12
Density (g/cm^3^)	2.60–2.8
Tensile strength (MPa)	3500–4800
Elastic modulus (GPa)	85–95
Elongation at break (%)	2.5–3.2

**Figure 3:**
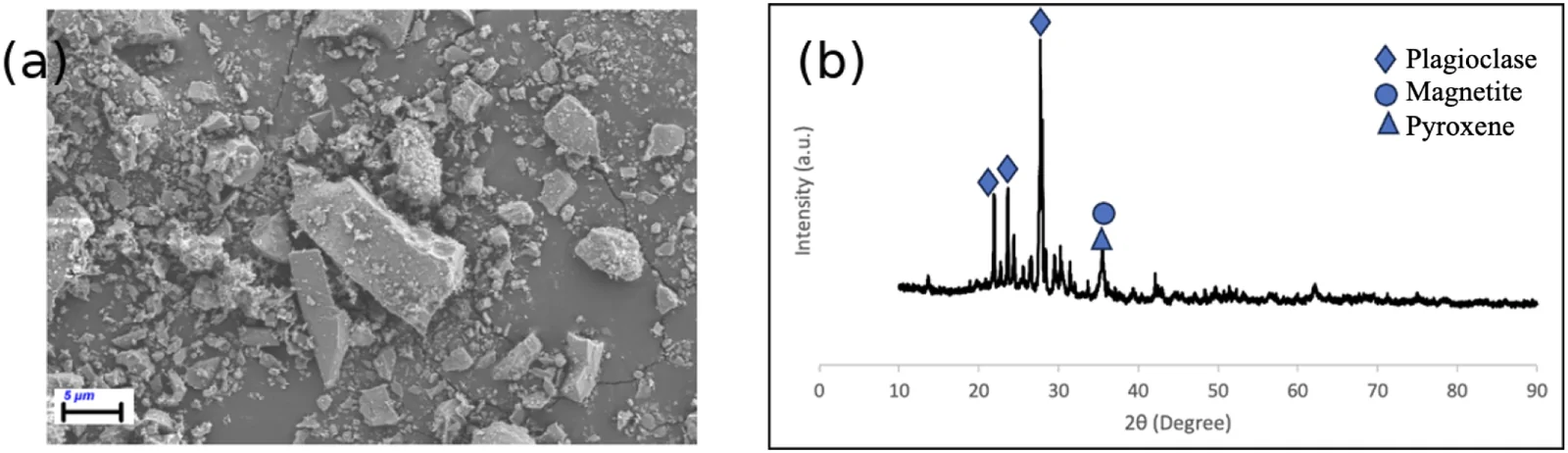
Microstructural and mineralogical characterization of volcanic ash (VA): (a) SEM image; (b) XRD pattern.

Figure 3 presents the morphological and mineralogical characteristics of volcanic ash (VA). As observed in Figure 3a, the SEM micrograph shows that VA particles exhibit irregular, angular shapes with rough, fractured surfaces. Such morphological features increase the particles’ specific surface area and may enhance their reactivity in cementitious systems. In addition, the presence of fine particles surrounding larger grains suggests a heterogeneous particle-size distribution that may enhance particle packing. Figure 3b shows the XRD pattern of VA, where several crystalline phases, including plagioclase, magnetite, and pyroxene, were identified from their characteristic diffraction peaks. Phase identification was performed by comparing the measured diffraction pattern with reference data from the International Center for Diffraction Data (ICDD) Powder Diffraction File (PDF) database. The major diffraction peaks observed at approximately 21–23°, 27–29°, and 34–36° (2θ) were attributed to plagioclase, magnetite, and pyroxene phases, respectively. In addition to these crystalline reflections, a relatively broad background hump indicates the presence of an amorphous glassy phase. This amorphous component is responsible for the pozzolanic reactivity of VA and can contribute to secondary C–S–H formation and matrix densification in cement-based materials.

### 2.2 Mixing Procedures

All concrete mixtures were produced in a laboratory pan mixer following a standardized mixing procedure to ensure homogeneous blending. Each mixture was proportioned to achieve a target compressive strength class of C30/37, in accordance with the relevant standard. Initially, the dry constituents, including CEM I 42.5 R cement, natural aggregates, and recycled coarse aggregates, were placed into the mixer and blended for approximately 2 min to obtain a uniform dry mix.

Prior to addition, the mixing water and the polycarboxylate ether–based superplasticizer (SP) were premixed to ensure complete dispersion in the mixing water. For the control mixture and plain concrete mixes without fibers, the SP dosage was kept constant and expressed as a percentage of the total binder mass. In the mixtures incorporating basalt (BA) fibers, the SP dosage was slightly increased with increasing fiber content to compensate for the expected loss of workability associated with fiber inclusion.

After preparation of the SP–water solution, it was gradually introduced into the dry mixture while the mixer was running, and wet mixing was continued for approximately 1 min. Once the fresh mixture reached a cohesive state, the BA fibers were added manually as the final component. The fibers were slowly introduced in small portions directly into the rotating drum to prevent the formation of fiber clumps and to promote uniform fiber dispersion throughout the concrete matrix. Following fiber addition, mixing was continued for an additional 2 min to ensure complete homogenization.

The fresh concrete was then cast into molds in two layers, with each layer compacted using a vibrating table to eliminate entrapped air. Specimens were demolded after 24 h and subsequently cured in water at 20 ± 2 °C until the 28-day testing age. The detailed mixture proportions, expressed in kilograms per cubic meter of concrete, are presented in Table 3.

**Table 3:** Mix Proportions of K0–K6 for 1 m^3^ of Concrete

Mix ID	VA (%) (binder)	BA fibers (% vol.)	Cement (kg/m^3^)	VA (kg/m^3^)	Water (kg/m^3^)	Water-to-binder ratio	SP (%) (binder)	0–5 mm (kg/m^3^)	5–12 mm (kg/m^3^)
VA0BA0	0	0.00	350	0	175	0.50	0.15	750	1050
VA5BA0	5	0.00	332.5	17.5	175	0.50	0.19	750	1050
VA10BA0	10	0.00	315	35	175	0.50	0.23	750	1050
VA15BA0	15	0.00	297.5	52.5	175	0.50	0.25	750	1050
VA10BA0.25	10	0.25	315	35	175	0.50	0.36	750	1050
VA10BA0.50	10	0.50	315	35	175	0.50	0.54	750	1050
VA10BA1.00	10	1.00	315	35	175	0.50	1.00	750	1050

### 2.3 Testing Methods

Mechanical and durability tests were conducted to evaluate the performance of the RAC mixtures incorporating BA fibers.

Water absorption (WA) and compressive strength (CS) tests were performed on cube specimens measuring 100 × 100 × 100 mm. WA measurements were carried out in accordance with ASTM C642,^31^ while CS testing followed the procedures specified in TS EN 12390-3.^32^ The unit weight of the mixtures was also determined using the same cube specimens.

Flexural strength (FS) was evaluated using prismatic specimens with dimensions of 100 × 100 × 400 mm under a three-point loading configuration in accordance with TS EN 12390-5.^33^ The span length and loading rate were selected to meet the standard’s requirements.

Chloride ion penetrability was assessed by the Rapid Chloride Permeability Test (RCPT) in accordance with ASTM C1202.^34^ For this purpose, 100 mm × 200 mm cylindrical samples were prepared, and 50 mm-thick discs were cut from the cured cylinders. The total charge passed (Coulombs) during the test period was recorded and used as an indicator of chloride permeability. The specimen preparation procedure and the Rapid Chloride Permeability Test (RCPT) setup are illustrated in Figure 4.

**Figure 4:**
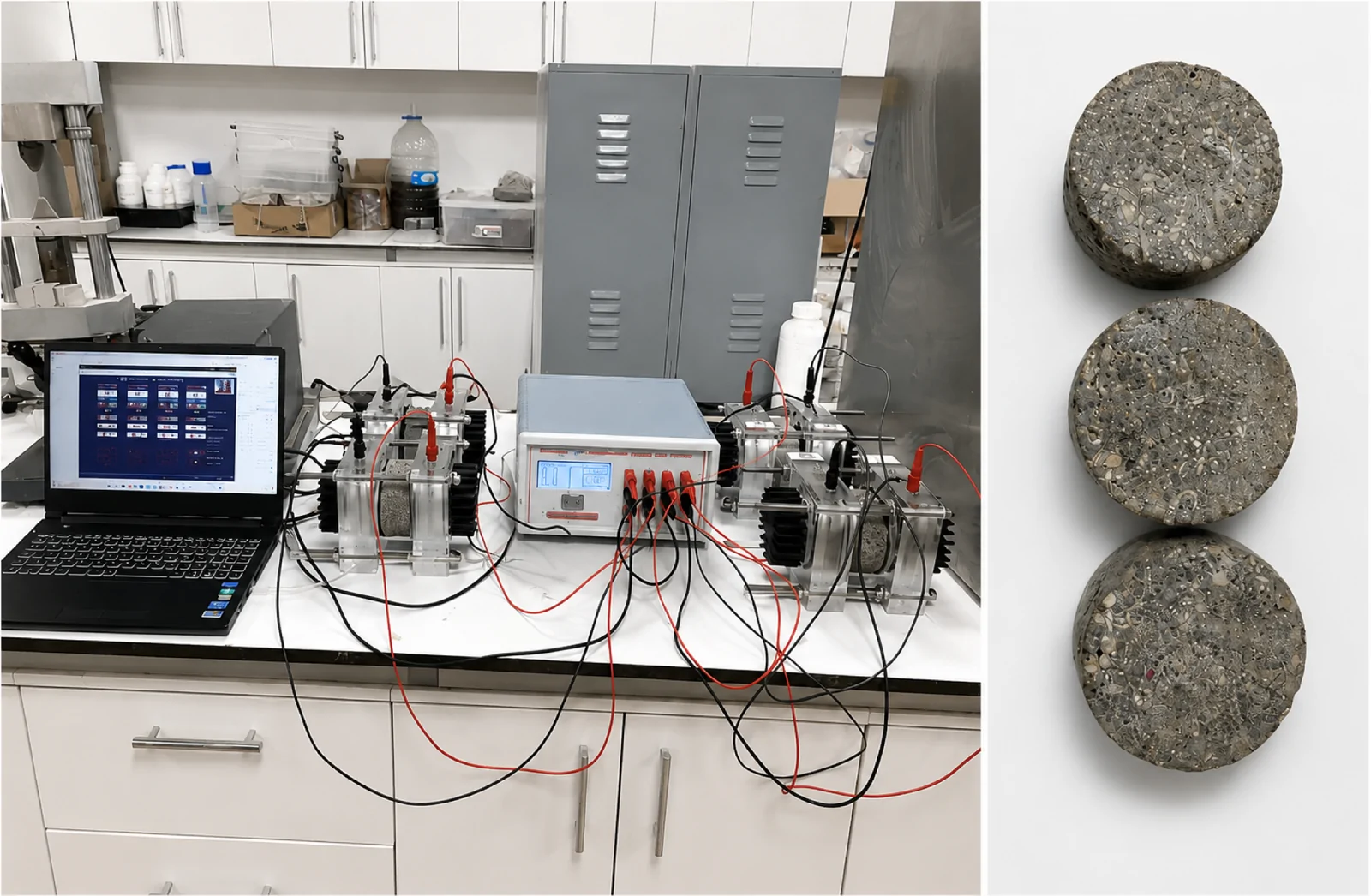
Specimen preparation and configuration of the Rapid Chloride Permeability Test (RCPT).

For each mixture and test parameter, three specimens (n = 3) were prepared and tested. The reported values represent the arithmetic mean of the three measurements, while the error bars shown in the figures correspond to the associated standard deviations. All regression analyses were performed using the mean values obtained from the investigated mixtures. All tests were performed under controlled laboratory conditions to minimize variability and maintain consistency throughout the experimental program.

Microstructural investigations were conducted at the Van Yüzüncü Yıl University Application and Research Center using a ZEISS EVO LS10 scanning electron microscope (SEM). Small fragments were extracted from the interior portions of the hardened concrete specimens after mechanical testing to minimize surface contamination and edge effects. Representative SEM micrographs were then obtained from these fragments and from the volcanic ash particles.

SEM images were acquired at different magnifications depending on the microstructural feature under investigation. Concrete specimens were examined at magnifications of approximately 250× and 500×, corresponding to scale bars of 100 and 20 μm, respectively, to evaluate matrix morphology, pore distribution, and fiber–matrix interactions. Volcanic ash particles were examined at a magnification of approximately 1000× with a scale bar of 5 μm.

The acquired SEM images were subsequently analyzed using ImageJ software to evaluate matrix porosity and void distribution. Prior to analysis, the images were converted to grayscale and processed using a consistent threshold-based image segmentation procedure. Void regions were identified based on their darker contrast relative to the surrounding cementitious matrix and converted into binary images for quantitative analysis. The segmented void areas were quantified as area fractions, and the identified void regions were highlighted in red to facilitate visualization and comparison among the mixtures. The same image-processing protocol was applied to all analyzed images to ensure consistency and reproducibility in the void quantification procedure.

## 3 Results and Discussion

### 3.1 Fresh and Hardened Density

Fresh density (FD) and hardened density (HD) values are presented in Figure 5. The results demonstrate a consistent reduction with increasing VA replacement and BA fiber content.

**Figure 5:**
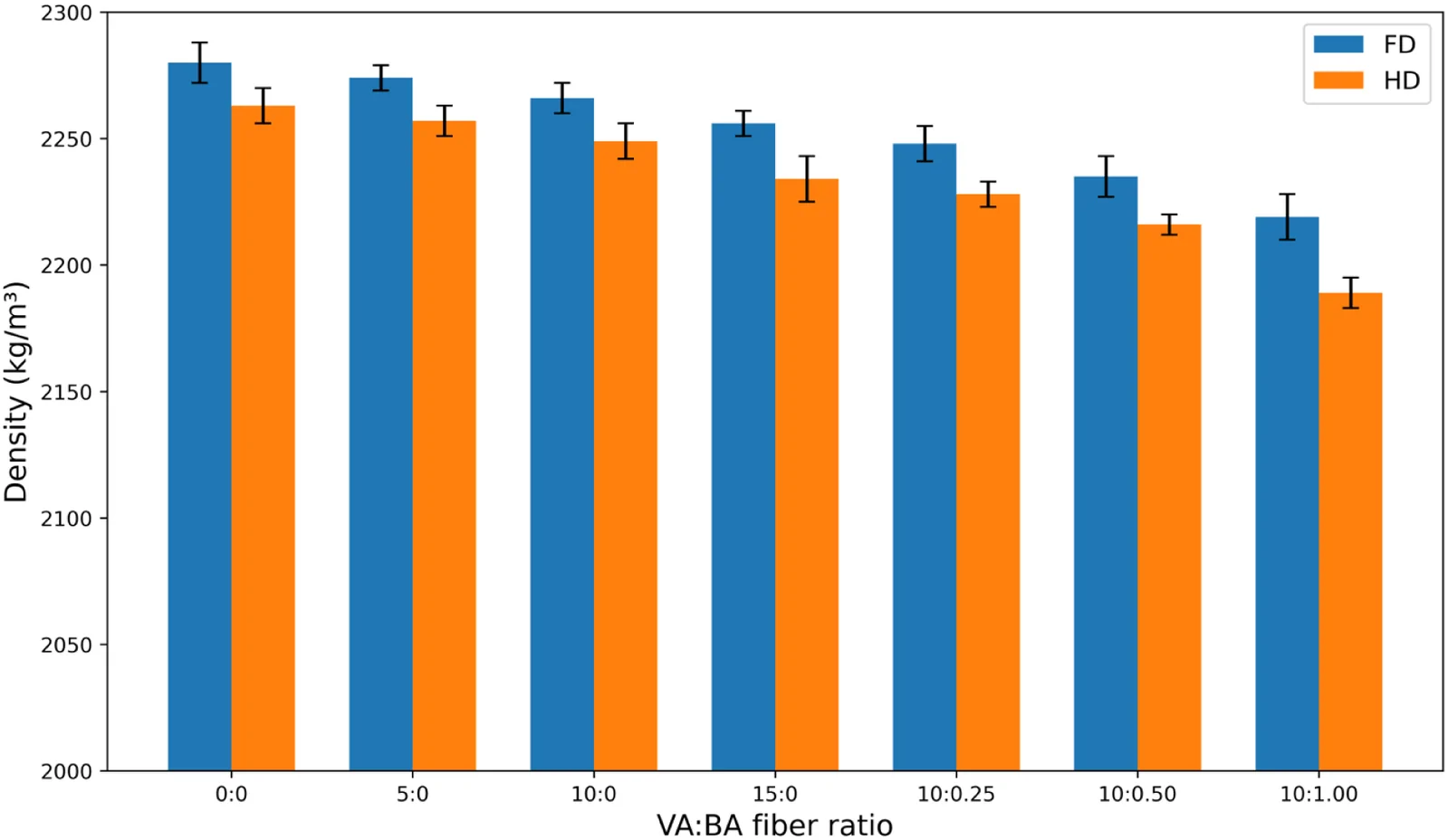
Variation of fresh density (FD) and hardened density (HD) of RAC mixtures with different VA replacement ratios and BA fiber volume fractions. The labels on the *x*-axis represent the VA replacement ratio (%) and BA fiber volume fraction (%), respectively.

The density results obtained for mixtures K0–K6 indicate that both volcanic ash (VA) replacement and basalt (BA) fiber incorporation systematically affected the fresh density (FD) and hardened density (HD) of the recycled aggregate concrete mixtures. Since the aggregate proportions and water-to-binder ratio remained constant across all mixtures, the observed density variations are mainly due to changes in the binder composition and fiber content.

The influence of VA replacement can first be observed in mixtures K0–K3, where the VA substitution ratio increased from 0% to 15%. In this group, FD gradually decreased from 2280 to 2256 kg/m^3^, while HD declined from 2263 to 2234 kg/m^3^. This reduction is primarily attributed to the lower specific gravity of VA compared with Portland cement.^35^ Partial replacement of cement with VA decreases the average density of the binder phase, thereby slightly reducing the total unit weight of the concrete mixtures.^36^ Because the aggregate quantities were kept constant, the density reduction mainly originated from the lower mass contribution of the modified binder system. Similar decreasing trends observed in both FD and HD further indicate that the density variation was mainly governed by the intrinsic material densities rather than by significant changes in the hardened microstructure.^37^

The influence of BA fibers became more pronounced in mixtures K2–K6, where the VA content was kept constant at 10% while the BA fiber volume fraction increased from 0% to 1%. In this series, FD decreased from 2266 to 2219 kg/m^3^, whereas HD declined from 2248 to 2189 kg/m^3^. Although basalt fibers possess relatively high intrinsic density,^38^ the incorporation of discrete fibers modifies the internal structure and packing characteristics of the composite material. The presence of fibers increases the number of fiber–matrix interfacial transition zones (ITZs), which may slightly disturb particle arrangement and contribute to localized void formation within the matrix.^39^ In addition, fiber incorporation partially replaces the cementitious matrix volume and alters the overall mass-to-volume balance of the composite system depending on the relative density differences between the fibers and the surrounding matrix phase.^40^ These mechanisms collectively contributed to the gradual reduction observed in both FD and HD values with increasing BA fiber content.

### 3.2 Water Absorption

Water absorption (WA) results are presented in Figure 6. The WA results of mixtures K0–K6 clearly demonstrate that both VA replacement and BA fibers volume fraction significantly influence the pore structure characteristics of the concrete. Since WA is directly related to total porosity and pore connectivity, the observed variations provide insight into how binder modification and fiber incorporation alter internal transport pathways.

**Figure 6:**
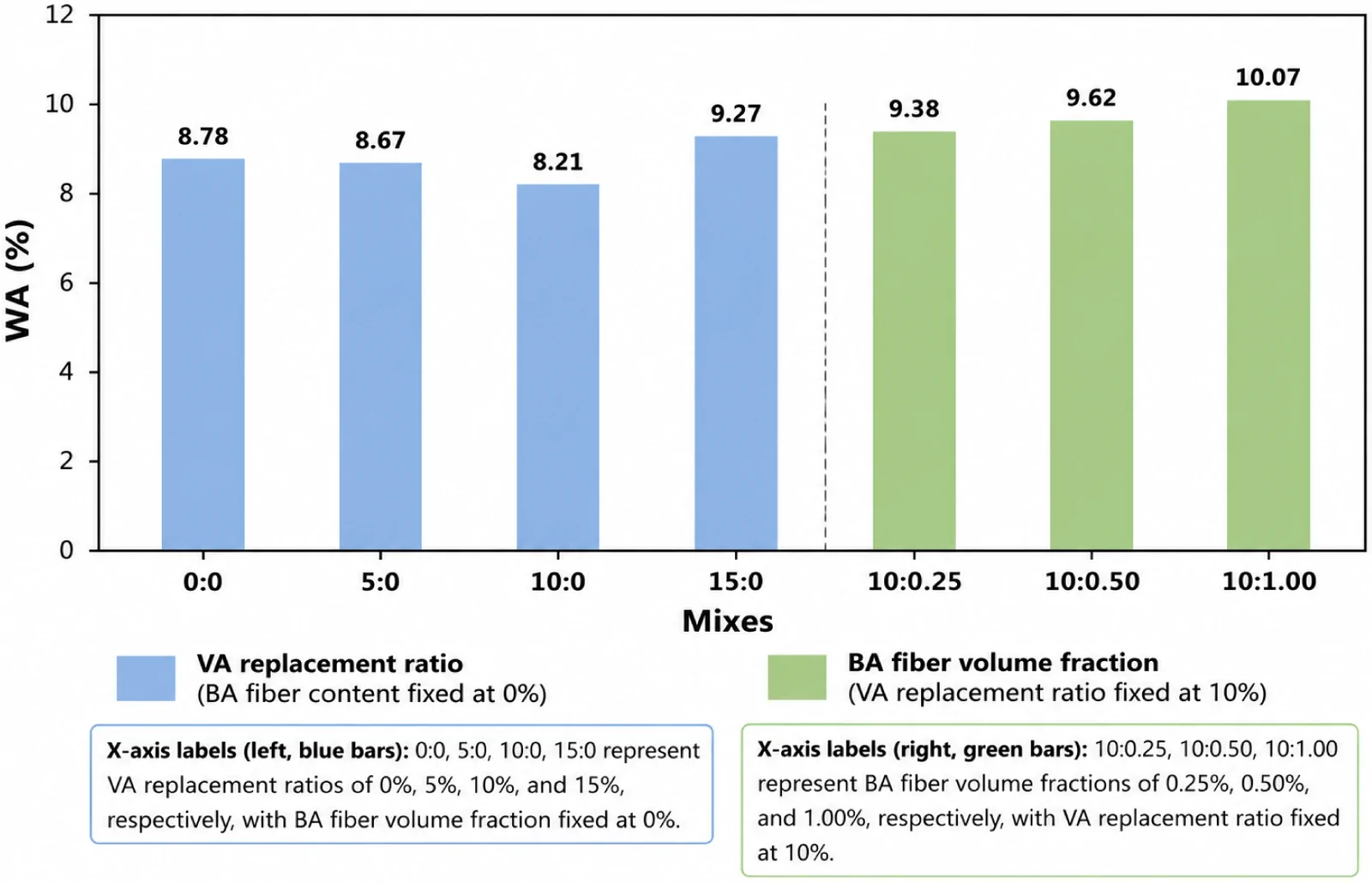
Water absorption (WA) of the investigated mixtures. The blue bars (0:0, 5:0, 10:0, and 15:0) represent volcanic ash (VA) replacement ratios of 0%, 5%, 10%, and 15%, respectively, with basalt fiber (BA) content fixed at 0%. The green bars (10:0.25, 10:0.50, and 10:1.00) represent BA fiber volume fractions of 0.25%, 0.50%, and 1.00%, respectively, with the VA replacement ratio fixed at 10%.

Considering first the influence of volcanic ash (VA) in mixtures K0–K3, where basalt (BA) fibers were not incorporated, the water absorption (WA) values gradually decreased from 8.78% (K0) to 8.67% (K1) and further to 8.21% (K2) as the VA replacement ratio increased from 0% to 10%. This reduction demonstrates that moderate VA incorporation significantly contributes to matrix refinement and pore-structure densification. Cai et al.^41^ reported that volcanic-based supplementary cementitious materials can improve the compactness and durability performance of cementitious systems through enhanced particle packing and pore refinement. Similarly, Kumar et al.^42^ emphasized that mineral-based fine materials improve hydration efficiency and matrix homogeneity, thereby reducing permeability-related transport mechanisms. In the present study, the fine VA particles likely filled microvoids between cement grains and hydration products, reducing pore continuity and limiting capillary transport pathways within the matrix. In addition, Jativa et al.^43^ highlighted that the amorphous silica-rich structure and pozzolanic reactivity of volcanic ash contribute significantly to densification behavior and reduced porosity in cementitious materials. The formation of additional secondary hydration products in the present mixtures likely contributed to the refinement of the internal microstructure and the reduction of fluid ingress pathways. Consequently, the capillary pore system became less interconnected, resulting in lower WA values and improved durability-related performance. Similar findings were also reported by Naseer et al.,^44^ who observed that moderate volcanic ash replacement levels improved water absorption behavior while maintaining satisfactory mechanical performance. These findings collectively indicate that moderate VA incorporation not only acts as a microfiller but also contributes to the development of a denser and more transport-resistant cementitious matrix.

However, when the VA content increased to 15% (K3), WA rose sharply to 9.27%, indicating that the beneficial influence of VA is limited to an optimum replacement threshold. Beyond this level, the reduction in Portland cement content likely exceeded the filler and pozzolanic contribution provided by VA, resulting in insufficient hydration product formation and weaker matrix densification. Consequently, the pore structure became more interconnected, facilitating moisture transport and increasing water absorption. Naseer et al.^44^ similarly reported that excessive volcanic ash incorporation may adversely influence matrix integrity and transport-related properties due to dilution effects and insufficient binder reactivity. Therefore, the present results suggest that excessive VA incorporation shifts the governing mechanism from pore refinement toward pore coarsening and increased transport connectivity within the cementitious system.

The influence of BA fibers became more pronounced when the VA content was fixed at 10% (K2–K6). Increasing the BA fiber volume fraction from 0% to 1% resulted in a continuous increase in WA from 8.21% (K2) to 9.38% (K4), 9.62% (K5), and 10.07% (K6). Although basalt fibers are known to improve crack resistance, toughness, and durability-related mechanical performance, Liu et al.^46^ emphasized that fiber incorporation simultaneously alters the internal microstructure and interfacial characteristics of cementitious composites. In the present study, the incorporation of discrete BA fibers likely introduced numerous interfacial transition zones (ITZs) between the fibers and the surrounding matrix, which may have acted as preferential pathways for water penetration when complete compaction and fiber dispersion were not fully achieved. Furthermore, Divyah et al.^45^ reported that increased void ratio and pore connectivity are closely associated with higher moisture transport and sorptivity in fiber-reinforced lightweight concrete systems. Similarly, higher BA fiber dosages in the present study likely promoted localized void formation, fiber agglomeration, and entrapped air within the matrix, thereby increasing pore connectivity and the potential for moisture transport. Therefore, while low-volume BA fibers may contribute positively to crack-bridging and postcracking resistance, excessive fiber incorporation can adversely affect transport-related durability parameters such as water absorption due to increased interfacial porosity and microstructural heterogeneity.

### 3.3 Rapid Chloride Permeability Test

Rapid chloride permeability test (RCPT) results are presented in Figure 7. The RCPT results for mixtures K0–K6 clearly indicate how VA replacement and the volume fraction of BA fibers influence the concrete’s chloride-ion penetration resistance. Since RCPT values are directly associated with pore connectivity and ionic transport pathways, the observed trends offer important insight into the evolution of the internal microstructure.

**Figure 7:**
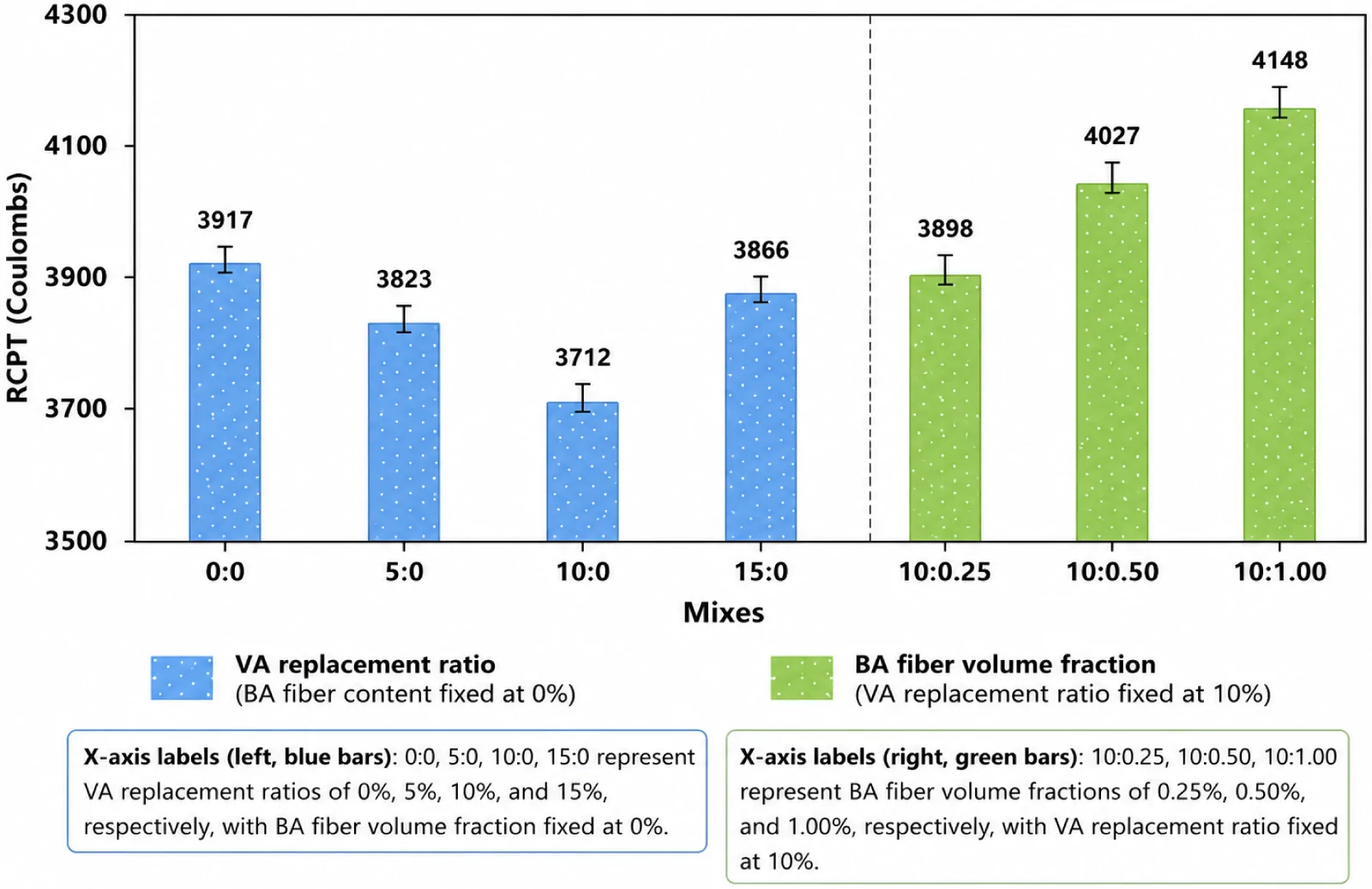
Rapid chloride permeability test (RCPT) results of the investigated mixtures. The blue bars (0:0, 5:0, 10:0, and 15:0) represent volcanic ash (VA) replacement ratios of 0%, 5%, 10%, and 15%, respectively, with basalt fiber (BA) content fixed at 0%. The green bars (10:0.25, 10:0.50, and 10:1.00) represent BA fiber volume fractions of 0.25%, 0.50%, and 1.00%, respectively, with the VA replacement ratio fixed at 10%. Error bars indicate the standard deviation of the measurements.

The influence of volcanic ash (VA) on chloride-ion penetration resistance can first be evaluated in mixtures K0–K3, in which basalt (BA) fibers were not incorporated. As the VA replacement ratio increased from 0% to 5% and then to 10%, the rapid chloride permeability test (RCPT) values decreased from 3917 C (K0) to 3823 C (K1), and then to 3712 C (K2). This reduction clearly indicates improved resistance to chloride-ion penetration at moderate VA replacement levels. Seyedsaleh et al.^47^ emphasized that chloride-ion transport in concrete is strongly governed by pore connectivity, binder characteristics, and the continuity of the internal transport network. In the present study, the fine-particle structure of VA likely improved particle packing and promoted secondary pozzolanic reactions, thereby refining the pore structure and reducing pore continuity within the cementitious matrix. Similarly, Ibrahim et al.^48^ reported that volcanic-based supplementary cementitious systems with refined pore connectivity significantly reduced chloride diffusivity and ionic mobility, due to the development of a denser, less interconnected transport network. The lower RCPT values observed in the present mixtures, therefore, suggest that moderate VA incorporation contributed to the formation of a more tortuous, transport-resistant microstructure, thereby limiting charge transfer during RCPT measurements. In addition, Kasaniya et al.^49^ highlighted that the reactivity and microstructural contributions of pozzolanic materials play a critical role in controlling chloride permeability and long-term durability. The lowest RCPT value obtained at 10% VA replacement indicates that this ratio provided an optimum balance between pore refinement, matrix densification, and sufficient cement hydration. Similar improvements in chloride penetration resistance associated with volcanic-based binders were also reported by Aguirre et al.,^50^ who observed significantly reduced charge-passed values in volcanic pozzolan-based systems due to enhanced transport resistance and denser binder structures.

However, when the VA replacement ratio increased to 15% (K3), the RCPT value increased to 3866 C. Although this value remained relatively close to that of the control mixture, the increase indicates that the beneficial influence of VA observed at moderate replacement levels diminished at excessive VA contents. Higher VA incorporation likely reduced the availability of primary cement hydration products due to the lower Portland cement content in the mixture. Consequently, the development of a sufficiently dense and continuous hydration structure became limited, leading to increased pore connectivity and facilitating ionic transport through the matrix. Kasaniya et al.^49^ similarly reported that the effectiveness of pozzolanic materials strongly depends on their reactivity and replacement level, and excessive substitution may adversely affect permeability-related performance due to insufficient matrix densification. Therefore, the present findings suggest that while moderate VA incorporation improves chloride resistance through pore refinement and transport restriction, excessive VA content may shift the governing mechanism toward increased ionic mobility and interconnected transport pathways.

The influence of BA fibers became more evident in mixtures K2–K6, where the VA replacement ratio was kept constant at 10% while the BA fiber volume fraction increased from 0% to 1%. In this series, RCPT values progressively increased from 3712 C (K2) to 3898 C (K4), 4027 C (K5), and 4148 C (K6). This gradual increase indicates a reduction in resistance to chloride-ion penetration with increasing BA fiber content. Guo et al.^51^ reported that although basalt fibers can improve the mechanical performance and crack resistance of concrete, excessive fiber incorporation may negatively influence chloride penetration resistance due to the formation of additional fiber–matrix interfacial transition zones (fiber-ITZs), increased porosity, and disturbed particle packing. A similar mechanism is likely responsible for the present findings. The incorporation of discrete BA fibers introduced numerous ITZ regions within the composite structure, which may have acted as preferential pathways for ionic transport when complete compaction and fiber dispersion were not fully achieved. Furthermore, higher fiber contents likely promoted localized void formation, entrapped air, and increased pore connectivity within the matrix, thereby facilitating charge transfer during RCPT measurements. These findings are also partially consistent with the observations of Zhang et al.,^52^ who demonstrated that the influence of basalt fibers on durability performance strongly depends on fiber dosage and microstructural evolution. Their study revealed that optimized basalt fiber incorporation can improve transport resistance and pore structure, whereas excessive or nonoptimized fiber content may increase permeability-related transport mechanisms by altering pore characteristics and interfacial structure. Therefore, the present results indicate that although low-volume BA fibers may contribute positively to crack-bridging and structural integrity, excessive BA incorporation can adversely affect chloride-ion penetration resistance because of increased microstructural heterogeneity and transport connectivity within the cementitious matrix.

Figure 8 illustrates the linear regression relationship between WA and RCPT for the investigated RAC mixtures. A strong positive correlation was observed (R^2^ = 0.849), indicating that the linear regression model provides a good fit to the relationship between water absorption and chloride penetrability in the investigated data set. The regression equation (y = 206.00x + 2029.53) suggests that RCPT tends to increase by approximately 206 coulombs for every 1% increase in WA. The high R^2^ value reflects the strength of the statistical association between these parameters and should not be interpreted as a direct measure of causal influence. The observed relationship is consistent with the role of pore connectivity and capillary continuity in governing moisture transport and chloride-ion penetration in cementitious materials.^53^ In RAC, the presence of recycled aggregates and associated ITZ significantly influences transport properties. A denser ITZ, promoted by VA incorporation, reduces microcrack formation and limits ion migration pathways,^54^ leading to lower WA and RCPT values. Conversely, mixtures with increased porosity or fiber–matrix interfacial discontinuities exhibit higher WA and, consequently, greater chloride penetrability.

**Figure 8:**
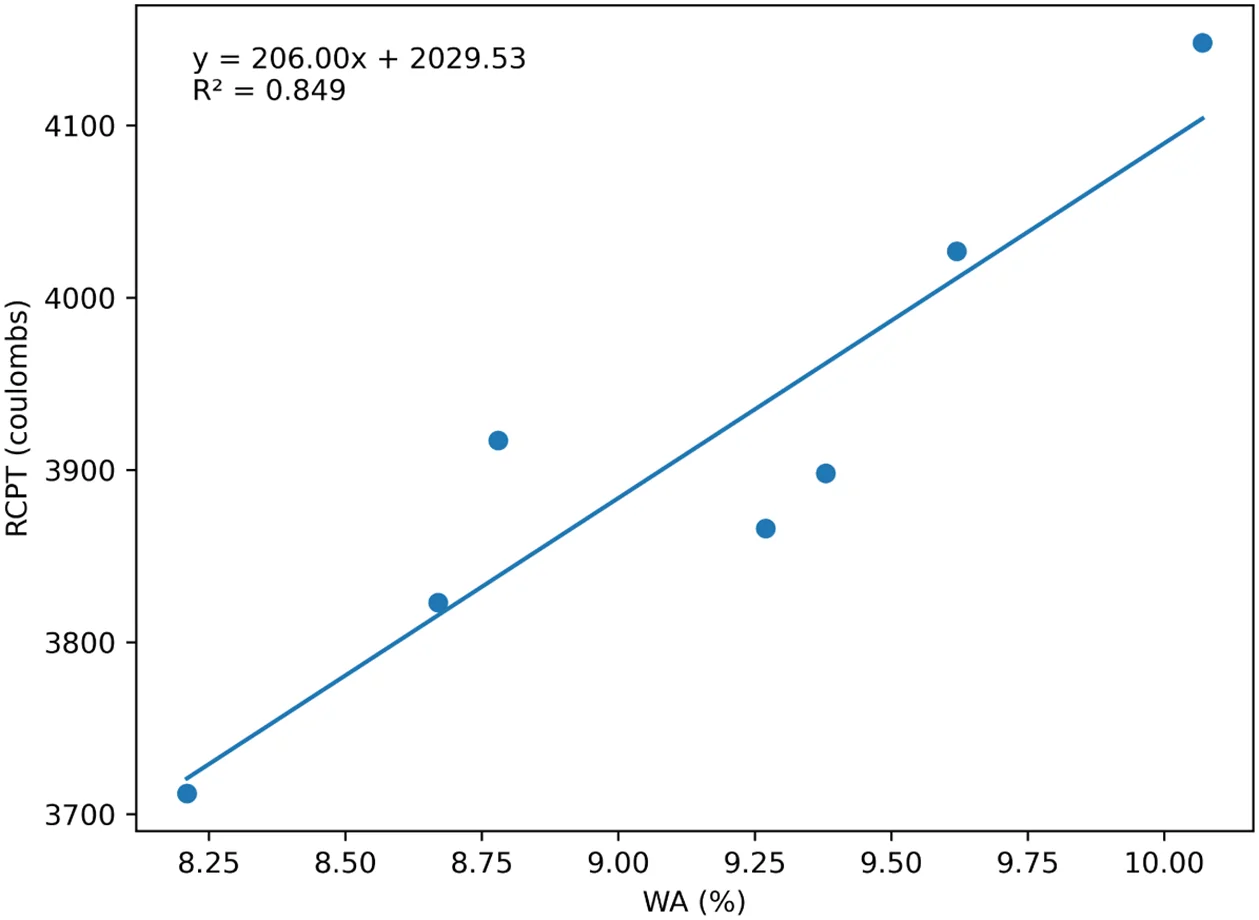
Relationship between WA and chloride penetrability (RCPT) in RAC mixtures.

### 3.4 Compressive Strength

Compressive strength (CS) results at 28 days are presented in Figure 9. The 28-day CS results of mixtures K0–K6 clearly demonstrate the influence of VA replacement and BA fiber volume fraction when evaluated relative to the control mixture (K0). Since K0 represents the reference system without VA and BA fiber, all strength variations are interpreted as percentage increases or decreases compared to this control.

**Figure 9:**
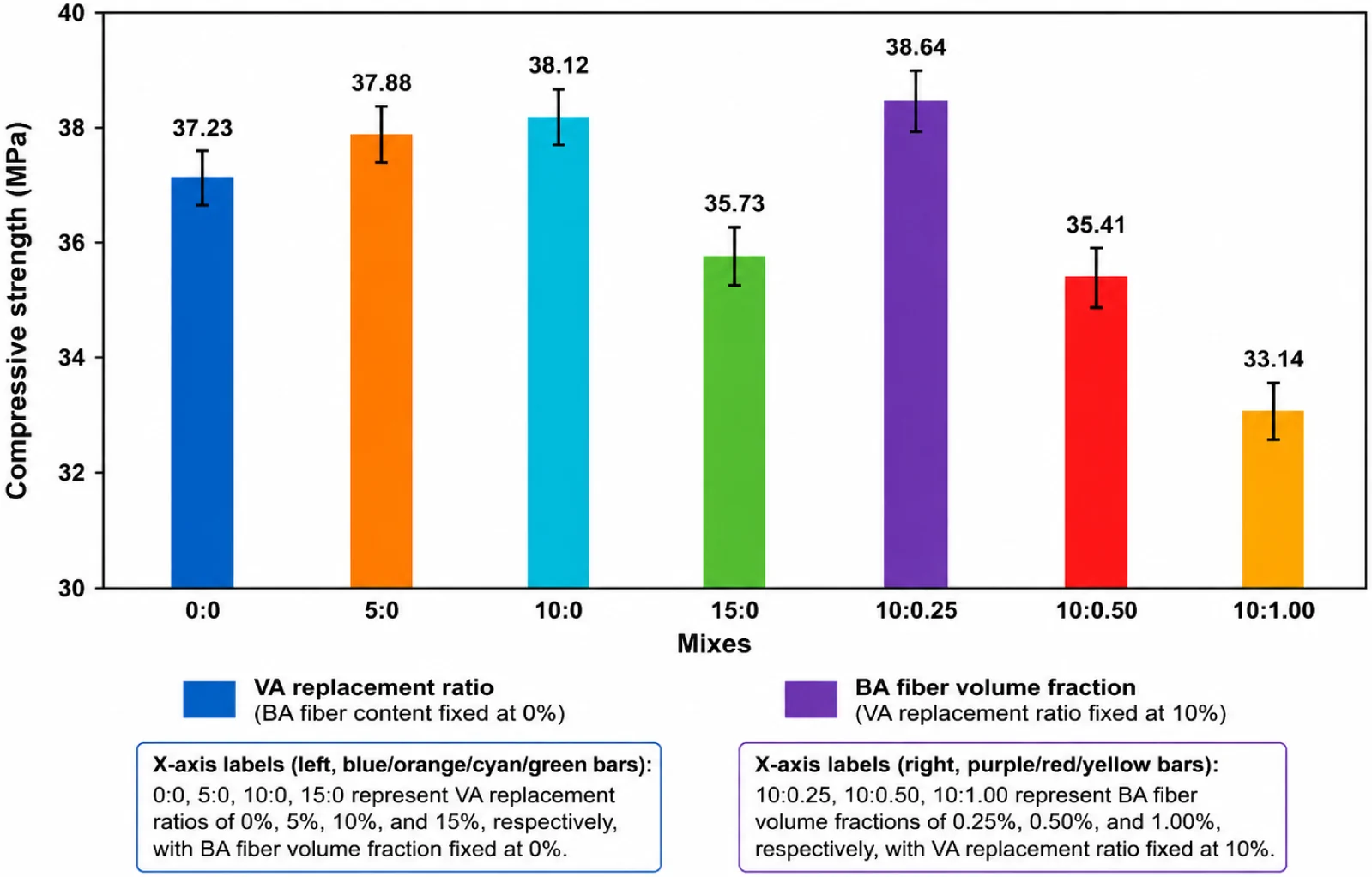
Compressive strength results of the investigated mixtures. The blue bars (0:0, 5:0, 10:0, and 15:0) represent volcanic ash (VA) replacement ratios of 0%, 5%, 10%, and 15%, respectively, with basalt fiber (BA) content fixed at 0%. The purple bars (10:0.25, 10:0.50, and 10:1.00) represent BA fiber volume fractions of 0.25%, 0.50%, and 1.00%, respectively, with the VA replacement ratio fixed at 10%. Error bars indicate the standard deviation of the measurements.

Considering first the influence of volcanic ash (VA) without basalt (BA) fiber incorporation (K1–K3), the compressive strength (CS) results demonstrated that moderate VA replacement improved the load-bearing performance of the recycled aggregate concrete mixtures. Mixture K1 containing 5% VA achieved a CS value of 37.88 MPa, corresponding to a 1.75% increase compared with the control mixture K0 (37.23 MPa). Similarly, increasing the VA replacement ratio to 10% (K2) further improved the CS value to 38.12 MPa, representing an enhancement of approximately 2.39% relative to K0. These improvements indicate that moderate VA incorporation enhances matrix densification and stress-transfer efficiency under compressive loading conditions. Karolina and Putra^55^ reported that volcanic ash-based mineral additions can improve compressive performance by enhancing particle packing and producing a denser cementitious structure. Similarly, Abdulazeez et al.^57^ observed that volcanic ash replacement levels around 10% provided optimum compressive performance due to the pozzolanic contribution of the ash and improved microstructural compactness. In the present study, the fine-particle structure of VA likely filled microvoids within the binder system and promoted secondary hydration reactions, thereby reducing internal defects and improving matrix continuity. These mechanisms collectively enhanced the stress distribution capability of the mixtures and contributed to improved compressive behavior. The present findings, therefore, suggest that moderate VA incorporation not only acts as a sustainable cement-replacement strategy but also contributes directly to the development of a denser and mechanically more stable cementitious matrix.

However, when the VA replacement ratio increased to 15% (K3), the CS value decreased to 35.73 MPa, corresponding to a 4.03% reduction relative to the control mixture. This reduction indicates that the beneficial contribution of VA diminished beyond the optimum replacement level. Excessive VA incorporation likely reduced the effective Portland cement content to a level insufficient to form a dense, continuous hydration network. Consequently, the dilution effect became dominant, limiting the formation of hydration products responsible for strength development and matrix integrity. Abdulazeez et al.^57^ similarly reported that although moderate volcanic ash incorporation improves strength performance, excessive replacement levels may adversely affect compressive behavior because of insufficient binder reactivity and reduced hydration-product formation. In addition, Bogas and Gomes^56^ emphasized that volcanic-based lightweight systems may exhibit reduced mechanical performance when increased porosity and insufficient matrix densification become dominant. Therefore, the present findings indicate that the mechanical performance of VA-containing mixtures depends strongly on achieving a balance between the beneficial microfiller/pozzolanic effects and the negative influence associated with excessive cement dilution.

The influence of BA fibers can be further evaluated by comparing the fiber-reinforced mixtures with the control mixture K0. The incorporation of 0.25% BA fiber (K4) increased the CS value to 38.64 MPa, representing an improvement of approximately 3.79% relative to K0. This result indicates that low BA fiber incorporation positively contributes to compressive performance by improving stress redistribution capacity and restricting the propagation of microcracks within the cementitious matrix. Yang et al.^58^ demonstrated that appropriate incorporation of basalt fiber can delay crack initiation, reduce localized damage intensity, and shift macrocrack development toward distributed microcracking, thereby improving the overall mechanical stability of concrete. A similar mechanism is likely responsible for the improved compressive behavior observed in the present study. The discrete BA fibers likely enhanced crack-bridging capacity and delayed localized crack propagation under compressive loading, thereby improving matrix integrity and load-transfer efficiency. These findings indicate that properly optimized BA fiber incorporation can improve the structural integrity of recycled aggregate concrete by controlling microcracks and enhancing stress-transfer mechanisms.

When BA fiber content increased to 0.50% (K5), CS decreased to 35.41 MPa, corresponding to a 4.89% reduction compared with the control mixture. At 1% BA fiber incorporation (K6), CS further declined to 33.14 MPa, representing an 11.00% reduction relative to K0. These significant reductions indicate that excessive BA fiber incorporation adversely affected the internal structural homogeneity of the mixtures. Higher fiber volume fractions likely introduced increased interfacial transition zone (ITZ) areas, localized void formation, and fiber agglomeration within the matrix, thereby generating stress concentration regions under compressive loading. John and Dharmar^59^ emphasized that the mechanical efficiency of basalt fibers strongly depends on fiber geometry, aspect ratio, and volume fraction, and excessive fiber incorporation may negatively influence matrix continuity and mechanical stability. Therefore, while low-volume BA fibers improved crack resistance and stress redistribution, excessive fiber incorporation increased internal heterogeneity and weakened the compressive resistance of the mixtures. These findings collectively demonstrate that the mechanical performance of fiber-reinforced recycled aggregate concrete is governed not only by fiber addition itself but also by the optimization of the fiber–matrix interaction and internal microstructural stability.

In Figure 10(a), a negative linear relationship between WA and CS was observed. Initially, the regression analysis of all experimental data yielded the equation y = −2.371x + 58.267, with a coefficient of determination (R^2^) of 0.592, indicating a moderate correlation between the two parameters. However, one mixture exhibited atypical behavior, showing relatively high WA despite achieving high CS. This behavior is attributed to the crack-bridging effect of low-volume BA fibers, which enhanced load transfer capacity while simultaneously increasing localized pore connectivity at the fiber–matrix interface. Therefore, the regression analysis was recalculated, excluding this outlier point, resulting in an improved correlation: y = −2.792x + 61.496, with R^2^ = 0.872. The revised regression confirms a stronger inverse relationship between WA and CS, demonstrating that increasing WA, associated with higher porosity and capillary void content, generally leads to lower compressive strength.^60,61^ For transparency and comparison, the original regression plot, including all experimental data, was preserved as an inset in Figure 10(a).

**Figure 10:**
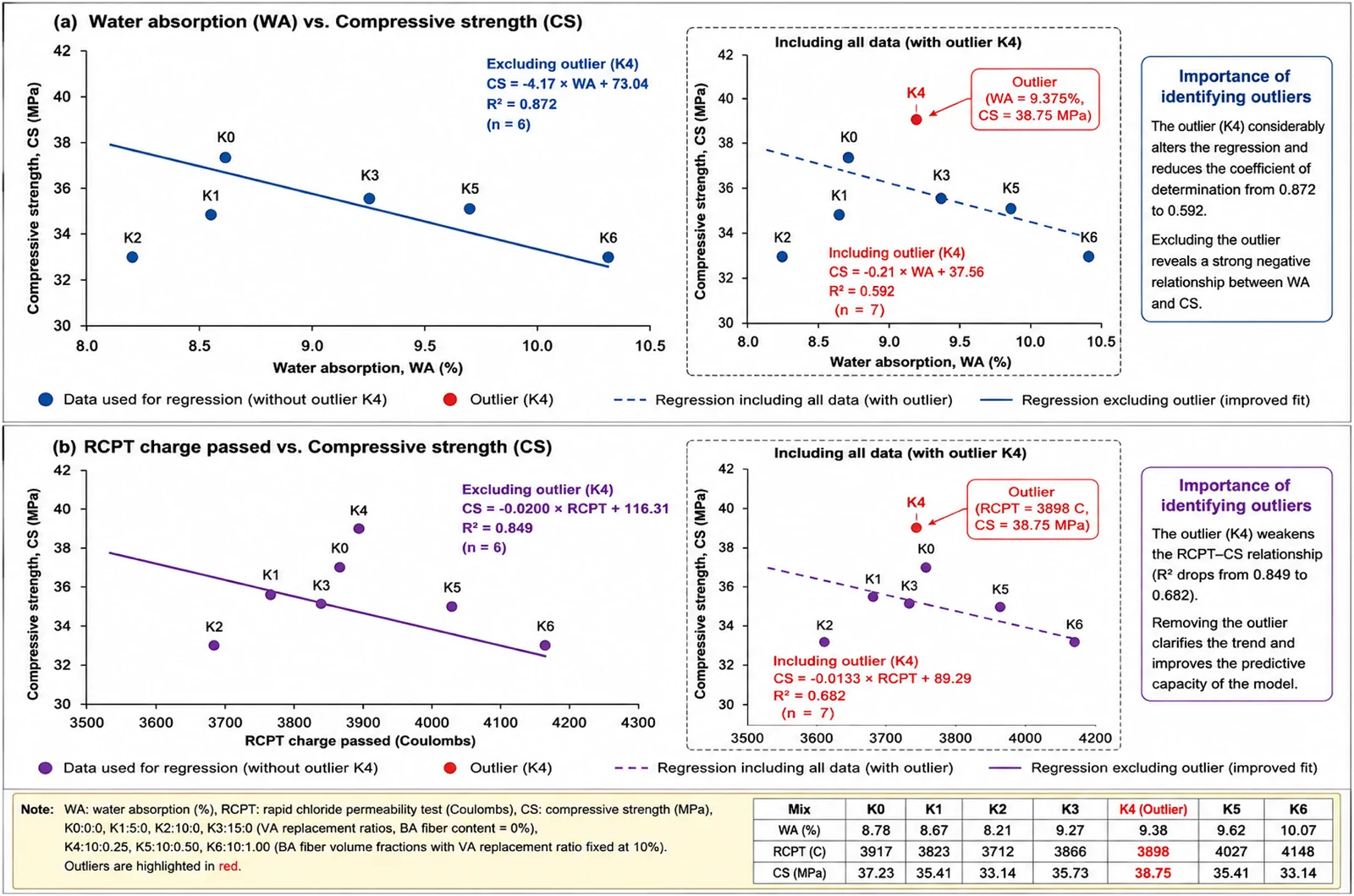
Effect of outlier identification on the regression relationships between (a) water absorption (WA) and compressive strength (CS), and (b) RCPT charge passed and compressive strength (CS). Figure 10 illustrates the linear regression relationships between compressive strength and (a) water absorption and (b) rapid chloride permeability (RCPT) values.

Similarly, Figure 10(b) shows the relationship between RCPT values and CS, with y = −0.011384x + 81.139 and R^2^ = 0.682. This result indicates that approximately 68.2% of the variation in CS is associated with changes in chloride permeability. Compared with the WA–CS relationship, the RCPT–CS correlation showed a more stable linear trend, with no clearly isolated outlier points; therefore, the original regression analysis was retained. The negative regression slope indicates that increased ion permeability is associated with reduced CS,^62^ highlighting the influence of pore connectivity and microstructural density on the mechanical and durability performance of the mixtures.

### 3.5 Flexural Strength

Flexural strength (FS) results at 28 days are presented in Figure 11.

**Figure 11:**
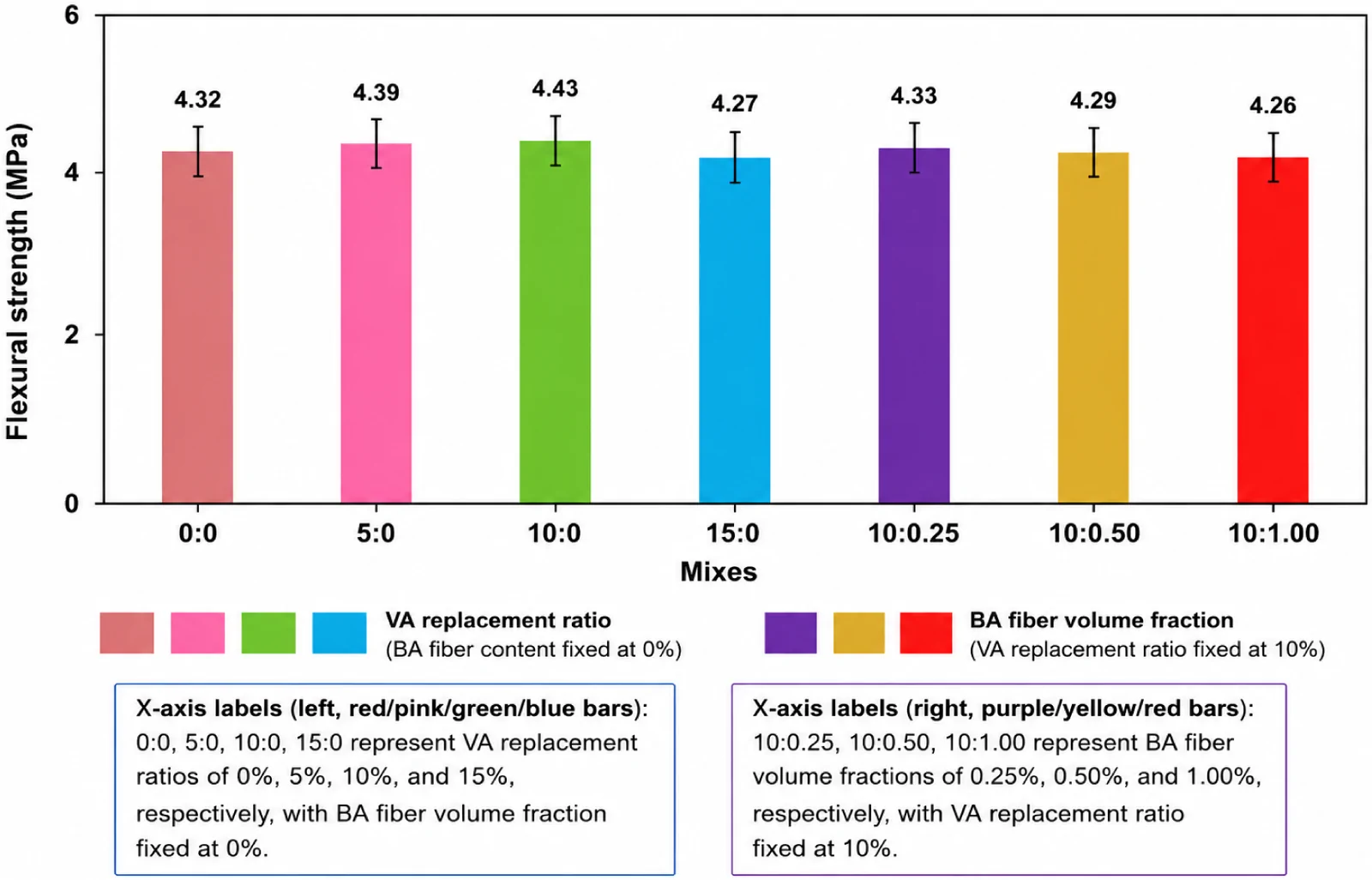
Flexural strength results of the investigated mixtures. The red, pink, green, and blue bars (0:0, 5:0, 10:0, and 15:0) represent volcanic ash (VA) replacement ratios of 0%, 5%, 10%, and 15%, respectively, with basalt fiber (BA) content fixed at 0%. The purple, yellow, and red bars (10:0.25, 10:0.50, and 10:1.00) represent BA fiber volume fractions of 0.25%, 0.50%, and 1.00%, respectively, with the VA replacement ratio fixed at 10%. Error bars indicate the standard deviation of the measurements.

The 28-day flexural strength (FS) results of mixtures K0–K6 demonstrated that, unlike compressive strength, FS did not exhibit a strong linear relationship with increasing volcanic ash (VA) replacement or basalt (BA) fiber volume fraction. When compared with the control mixture K0 (4.32 MPa), all FS values remained within a relatively narrow range of 4.26–4.43 MPa, indicating that peak flexural behavior was only moderately influenced by the investigated parameters. This finding suggests that the flexural response of the mixtures was governed not only by matrix densification but also by localized crack initiation mechanisms, interfacial characteristics, and tensile-zone heterogeneity within the composite structure.

Considering first the influence of VA without BA fiber incorporation (K1–K3), mixture K1 containing 5% VA achieved an FS value of 4.39 MPa, corresponding to an increase of approximately 1.62% relative to K0. Increasing the VA replacement ratio to 10% (K2) further increased the FS value to 4.43 MPa, representing an approximately 2.55% improvement over the control mixture. However, when the VA replacement ratio increased to 15% (K3), FS decreased to 4.27 MPa, corresponding to a reduction of approximately 1.16% relative to K0. These findings indicate that the influence of VA on FS was relatively limited and nonmonotonic compared with its effect on compressive strength. Presa et al.^63^ reported that volcanic ash-based pozzolanic systems can improve mechanical performance through enhanced hydraulic reactivity and matrix refinement, particularly at moderate replacement levels. Similarly, Zeyad et al.^65^ observed that volcanic pumice powder, when incorporated at optimized replacement ratios, improved flexural performance by enhancing matrix compactness and reducing transport-related defects. In the present study, the slight FS improvements observed at moderate VA contents were likely associated with improved particle packing and partial matrix densification within the tensile zone of the concrete. However, flexural behavior is highly sensitive to local heterogeneity, microcrack initiation, and the quality of the interfacial transition zone (ITZ) within the composite structure. Therefore, although moderate VA incorporation contributed to improved microstructural compactness, these improvements did not directly translate into substantial increases in peak flexural strength. At higher VA replacement levels, the reduction in effective cement content and the resulting dilution effect likely weakened matrix continuity and slightly reduced flexural resistance.

A similar trend was observed with BA fiber incorporation. When the VA replacement ratio was fixed at 10% (K2–K6), increasing the BA fiber volume fraction from 0% to 1% did not produce a systematic enhancement in FS. At 0.25% BA fiber (K4), FS was 4.33 MPa, corresponding to only +0.23% relative to K0, which is practically negligible within typical experimental variability. At 0.50% BA fiber (K5), FS decreased to 4.29 MPa (−0.69% vs K0), and at 1% BA fiber incorporation (K6), FS further declined to 4.26 MPa (−1.39% vs K0). These limited fluctuations indicate that BA fiber incorporation did not significantly alter the peak flexural capacity of the mixtures within the investigated dosage range. Alnahhal et al.^66^ emphasized that fiber reinforcement mechanisms primarily become effective after crack initiation, where fibers act as crack-bridging elements that improve tensile load redistribution, energy absorption, and postcracking resistance rather than substantially increasing the first peak flexural load. Similarly, Shafiq et al.^67^ reported that basalt fibers are generally more effective in improving postcracking ductility and toughness behavior than enhancing the initial peak flexural strength of cementitious composites. A similar mechanism is likely responsible for the present findings. Although BA fibers may have contributed to delaying crack propagation and improving structural integrity after crack formation, these effects were not strongly reflected in the peak FS values measured during the flexural test. Furthermore, excessive fiber incorporation may have increased localized heterogeneity, entrapped air, and fiber–matrix discontinuities, thereby limiting the efficiency of stress transfer within the tensile region. Overall, the percentage variations relative to K0 remained confined between +2.55% and −1.39%, demonstrating that peak FS behavior was largely matrix-controlled and only marginally affected by VA and BA fiber incorporation within the investigated mixture proportions.

Figure 12 presents the linear regression relationships between flexural strength and (a) water absorption, (b) rapid chloride permeability (RCPT), and (c) compressive strength. In Figure 12(a), a negative correlation between water absorption and flexural strength is observed, described by the equation y = −0.0879x + 5.131, with an R^2^ value of 0.776, indicating a strong linear relationship between the two parameters. The negative slope suggests that mixtures with higher water absorption generally exhibited lower flexural strength. Similarly, Figure 12(b) illustrates the relationship between RCPT and flexural strength, represented by y = −0.000367x + 5.763, with an R^2^ value of 0.676. The negative correlation indicates that mixtures with higher chloride-ion permeability generally exhibited lower flexural strength. This behavior suggests that increased pore connectivity and transport pathways contribute to reduced resistance against bending stresses.

**Figure 12:**
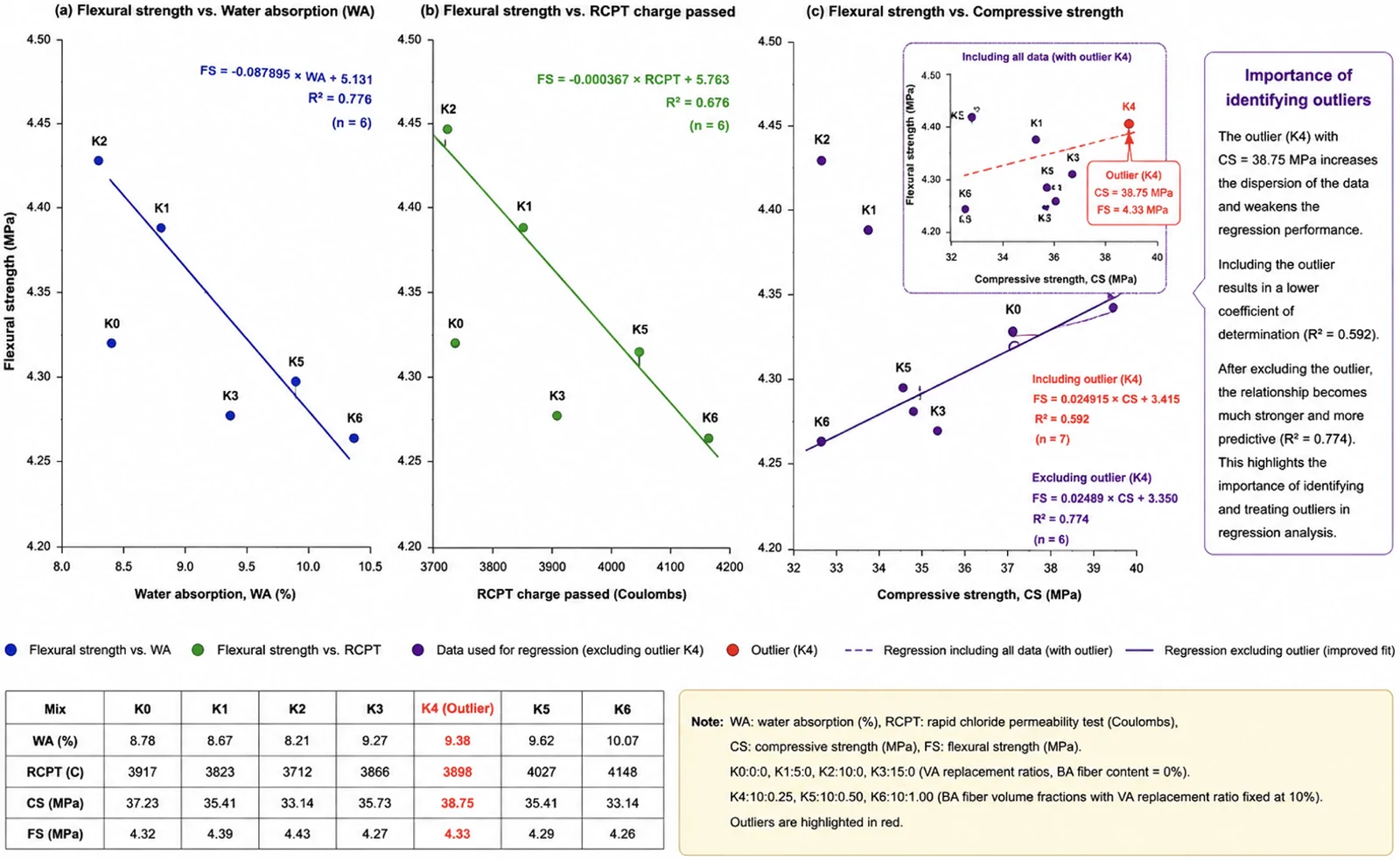
Linear regression relationships between flexural strength and (a) water absorption (WA), (b) rapid chloride permeability (RCPT), and (c) compressive strength (CS), illustrating the influence of the identified outlier on regression performance.

In Figure 12(c), the relationship between compressive strength and flexural strength is expressed by y = 0.0249x + 3.415, yielding an R^2^ value of 0.592. The positive slope confirms the expected compatibility between compressive and flexural performance, indicating that mixtures with higher compressive strength generally tend to exhibit improved flexural strength. However, the lower R^2^ value compared with those obtained for WA–FS and RCPT–FS suggests that durability-related parameters exhibited stronger correlations with flexural behavior than compressive strength within the investigated mixture system.

### 3.6 SEM Analysis

Scanning electron microscopy (SEM) was performed to investigate the microstructural characteristics of selected mixtures: K0 (control), K2 (10% VA), and K5 (10% VA + 0.50% BA fiber). Representative SEM micrographs of K0, K2, and K5 specimens are presented in Figure 13. These mixtures represent the reference condition, the optimum VA replacement level in terms of overall performance, and a fiber-reinforced system showing reduced mechanical efficiency, respectively.

**Figure 13:**
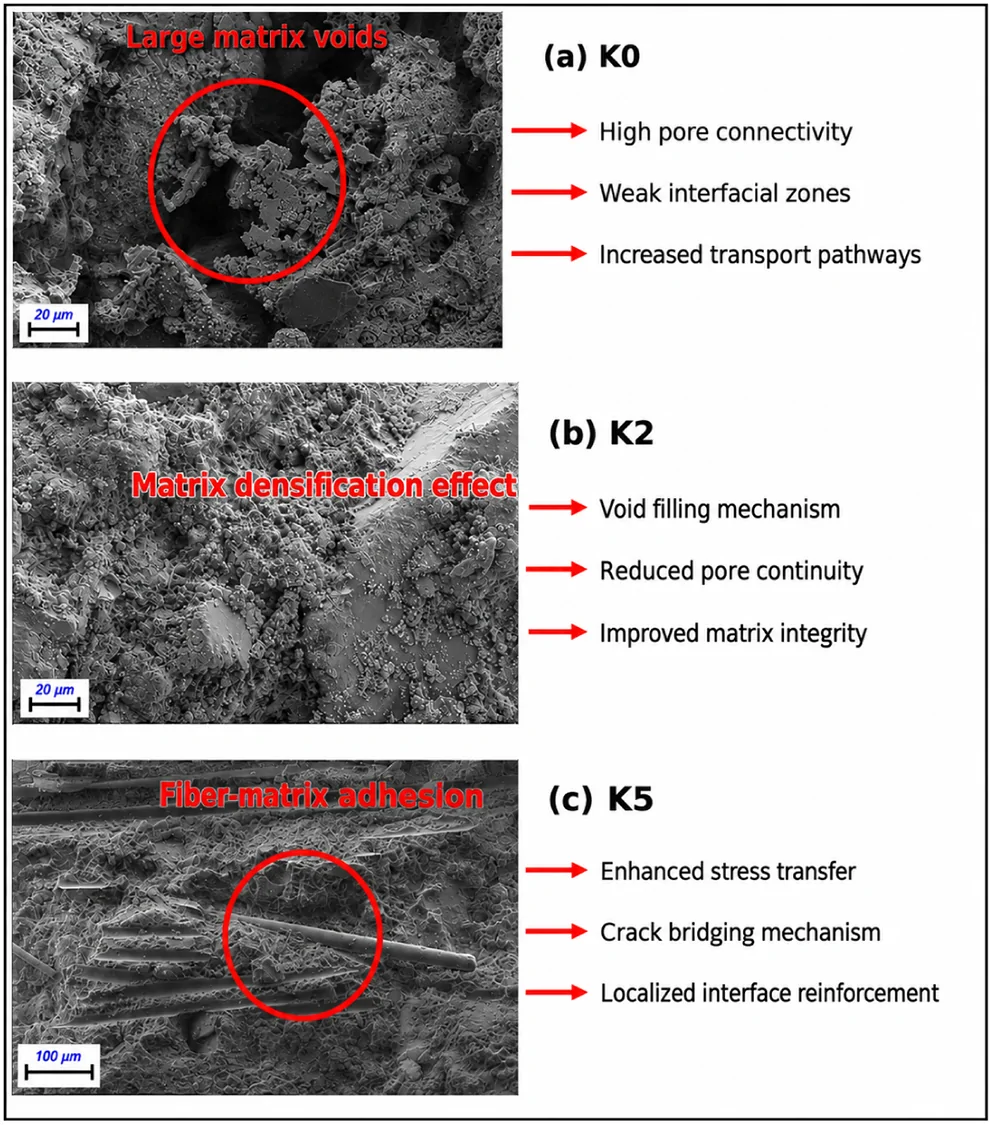
Representative SEM micrographs of (a) K0, (b) K2, and (c) K5 specimens.

SEM observations of the control mixture (K0) revealed a relatively heterogeneous and porous microstructure characterized by visible capillary pores, scattered microcracks, and locally discontinuous interfacial regions.^68^ The interfacial transition zone (ITZ) formed between the recycled aggregates (RA) and the surrounding cementitious paste appeared relatively weak and porous because of the presence of adhered old mortar on the RA surfaces.^69^ These adhered mortar layers contributed to irregular pore distribution, microvoid formation, and weak bonding characteristics within the matrix.^70^ In several regions, interconnected pores and interfacial gaps were clearly observed, indicating the existence of potential transport pathways facilitating fluid and ionic ingress.^71^

This microstructural condition is consistent with the experimentally measured durability and mechanical performance of the control mixture. The relatively high water absorption (WA) value of 8.78% and RCPT value of 3917 coulombs indicate elevated pore connectivity and increased ionic permeability within the matrix. Similarly, the baseline compressive strength (CS) value of 37.23 MPa reflects the adverse influence of porous ITZ regions and weak aggregate–paste bonding on load-transfer efficiency.^72^ Overall, the SEM findings confirm that pore continuity, microcrack distribution, and ITZ deficiencies were the dominant factors governing the transport and mechanical behavior of the control recycled aggregate concrete mixture.

In contrast, the K2 mixture containing 10% VA exhibited a noticeably denser, more homogeneous microstructure compared with the control mixture. SEM observations revealed improved particle packing, a more compact cementitious matrix, and fewer visible capillary voids. Feng et al.^73^ reported that volcanic ash-based systems can significantly improve microstructural compactness through filler and alkali-activation-related refinement mechanisms, thereby enhancing matrix continuity and reducing internal heterogeneity. Similar behavior was observed in the present study, where the incorporation of 10% VA promoted a more uniform distribution of hydration products within the matrix. In addition, the interfacial transition zone (ITZ) between the recycled aggregates and the surrounding paste appeared more continuous and tightly bonded. Santillán et al.^74^ emphasized that improved ITZ continuity plays a critical role in controlling durability-related transport properties and crack propagation in recycled aggregate systems. The denser ITZ structure observed in K2 likely contributed to improved stress transfer and reduced permeability-related transport mechanisms. Furthermore, Hou et al.^75^ reported that enhanced interfacial adhesion and reduced pore connectivity significantly improve the multiscale performance of recycled aggregate composites. In the present study, hydration products appeared to partially fill microvoids and reduce pore interconnectivity within the matrix, thereby limiting ionic and fluid transport pathways. Microcracks were also less pronounced and appeared more effectively restrained by the surrounding dense matrix. Tahwia et al.^76^ similarly observed that volcanic-powder-based sustainable cementitious systems can reduce the tendency toward internal cracking and improve microstructural integrity by enhancing matrix densification. These microstructural improvements correlate directly with the reduced WA (8.21%, 6.49% lower than K0) and reduced RCPT (3712 coulombs, 5.23% lower than K0) values obtained for K2. In parallel, CS increased to 38.12 MPa, representing a 2.39% improvement relative to the control mixture. Therefore, the SEM findings confirm that moderate VA incorporation improves matrix compactness, restricts transport connectivity, and enhances both durability and mechanical performance.

The K5 mixture (10% VA + 0.50% BA fiber) exhibited distinct microstructural characteristics associated with fiber–matrix interaction. Although the matrix still showed evidence of VA-induced refinement, SEM observations demonstrated that BA fibers actively contributed to crack-bridging mechanisms within the composite structure. Hong et al.^77^ reported that strong fiber–matrix bonding and interfacial adhesion play a critical role in improving stress redistribution and limiting crack propagation in fiber-reinforced cementitious composites. A similar mechanism was observed in the present study, in which BA fibers appeared to transfer stresses across localized microcracks and partially restrain crack opening in the matrix. The ITZ surrounding the fibers showed partial densification; however, localized interfacial discontinuities and microvoids were occasionally observed near the fiber surfaces. Wang et al.^78^ emphasized that basalt fibers may simultaneously improve crack resistance while introducing localized interfacial heterogeneity depending on fiber distribution and exposure conditions. In several regions of the present SEM images, BA fibers acted as physical barriers that interrupted crack propagation and partially restricted pore continuity. Similarly, Liu et al.^79^ reported that basalt fibers can improve matrix stability and reduce crack development through synergistic interaction with the surrounding cementitious structure, although excessive interfacial regions may alter local pore distribution. Therefore, the microstructural behavior observed in K5 reflects a balance between beneficial crack-bridging effects and localized interfacial heterogeneity associated with fiber incorporation.

To further quantify the microstructural differences observed in the SEM images, an ImageJ-based void segmentation analysis was performed, as presented in Figure 14. One representative SEM micrograph was selected from each investigated mixture (K0, K2, and K5) and analyzed using ImageJ software (Version 1.54, National Institutes of Health, Bethesda, MD, USA). The selected images were considered representative of the overall microstructural characteristics observed in the specimens.

**Figure 14:**
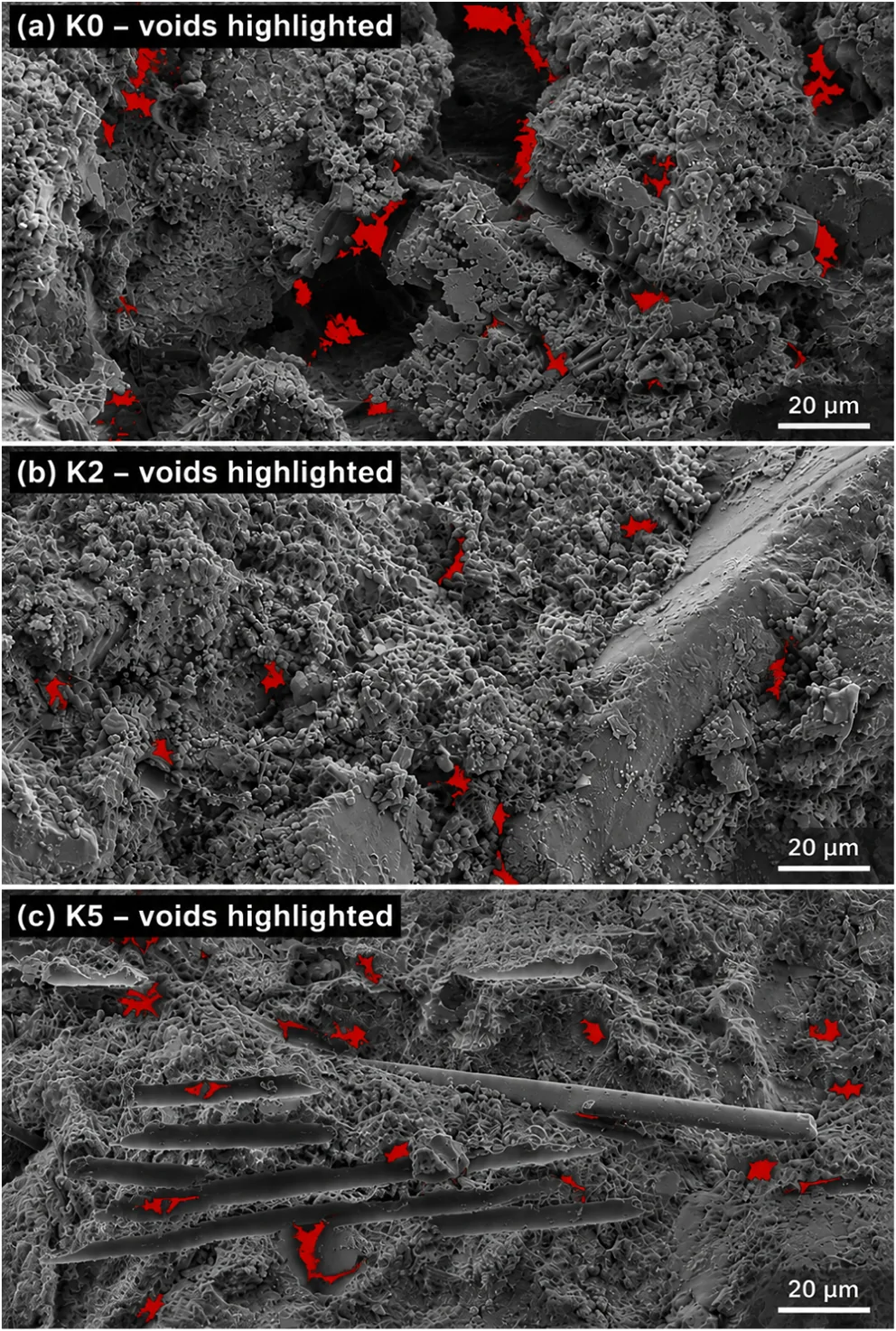
SEM micrographs showing the detected voids (red) in (a) K0, (b) K2 (10% VA), and (c) K5 (10% VA + 0.50% BA fiber). The void area fractions were 23.82%, 11.66%, and 14.43%, respectively.

The SEM micrographs were first converted to grayscale and contrast-enhanced using adaptive histogram equalization to improve phase discrimination. A consistent threshold value was applied to all images to distinguish void regions from the solid cementitious matrix. The resulting binary images were subsequently processed using morphological filtering to remove noise and isolated artifacts. Scale calibration was performed using the SEM scale bars, enabling conversion from pixel dimensions to real-scale measurements. The detected void regions were highlighted in red, and the void area fraction (%) was calculated as the ratio of the total detected void area to the total analyzed image area.

The quantitative ImageJ analysis revealed that the control mixture K0 exhibited the highest void area fraction of 23.82%, confirming its relatively porous and heterogeneous microstructure. In contrast, the K2 mixture containing 10% VA showed the lowest void area fraction, 11.66%, corresponding to a 51.05% reduction relative to K0. This result quantitatively verifies the matrix densification effect associated with the filler effect and pore-refinement mechanisms provided by volcanic ash. The K5 mixture (10% VA + 0.50% BA fiber) exhibited a slightly higher void area fraction of 14.43%. Although basalt fibers contributed to crack-bridging and crack-arresting mechanisms, localized interfacial voids formed around certain fiber–matrix interaction zones, resulting in a moderate increase in void content relative to K2. Nevertheless, the void area fraction of K5 remained 39.42% lower than that of the control mixture, indicating that the combined use of volcanic ash and basalt fibers still improved the overall microstructural compactness of the recycled aggregate concrete. It should be noted that the ImageJ-derived void area fractions represent comparative two-dimensional indicators of pore distribution rather than absolute porosity values.

## 4 Life Cycle Assessment (LCA)

### 4.1 Goal and Scope Definition

The primary objective of this life cycle assessment (LCA) was to evaluate the environmental performance of concrete mixtures incorporating volcanic ash (VA) and basalt (BA) fiber and to quantify the influence of cement replacement and fiber addition on selected environmental impact indicators. Unlike full building-scale assessments, the present study was designed as a comparative material-level analysis focusing specifically on the environmental implications of mixture modification and constituent selection. Serres et al.^80^ emphasized that comparative LCA approaches are particularly suitable for evaluating sustainable concrete systems because they enable consistent comparisons between mixtures with similar functional performance while isolating the environmental contributions of individual constituents.

A cradle-to-gate system boundary was adopted in the present study.^80^ Accordingly, the assessment included (i) raw material production processes associated with cement, VA, aggregates, superplasticizer, and BA fibers, and (ii) transportation of all constituents to the batching facility located in Van, Türkiye. Construction-stage activities, service-life performance, maintenance processes, and end-of-life scenarios were excluded because the primary focus of this research was the material-level environmental performance of the developed concrete mixtures. Bennett et al.^81^ similarly highlighted that the environmental influence of supplementary cementitious materials is most effectively quantified through mixture-level comparisons, particularly when evaluating reductions in cement-related emissions.

The functional unit was defined as 1 m^3^ of concrete, and all material quantities were normalized accordingly to ensure direct comparison among mixtures containing different VA and BA fiber contents. This approach enabled a consistent evaluation of the environmental influence associated with mixture modification independent of structural-scale considerations. The inventory modeling was performed using GaBi software (Sphera Solutions GmbH, Germany), and background inventory data were obtained from the GaBi database. The impact assessment focused on Global Warming Potential (GWP),^82^ Acidification Potential (AP),^83^ and Eutrophication Potential (EP).^83^ These impact categories were selected because they represent the most relevant environmental burdens associated with cementitious material production, transportation intensity, and energy consumption processes. Park et al.^82^ demonstrated that transportation and processing conditions can significantly influence GWP, AP, and EP values in sustainable concrete systems, whereas Colangelo et al.^83^ emphasized the importance of evaluating environmental trade-offs when alternative binder systems and recycled materials are incorporated into concrete production.

Transportation distances were determined based on realistic supply chain assumptions, including long-distance road transport for cement, regional transport for VA and aggregates, and combined sea–road transport for BA fibers imported from China. For imported BA fibers, the transportation chain consisted of marine freight from China to Mersin Port, Türkiye, followed by domestic road transportation from Mersin to Van. These assumptions enabled the simultaneous evaluation of both the environmental benefits of locally sourced VA use and the additional environmental burdens associated with globally sourced BA fibers. Therefore, the adopted LCA framework not only quantifies the environmental impact of modifying the mixture but also highlights broader sustainability trade-offs between the use of locally sourced supplementary cementitious materials and the use of imported high-performance fiber reinforcement in sustainable concrete production systems.

### 4.2 Life Cycle Inventory (LCI) and Mix Normalization

The life cycle inventory (LCI) was established based on the mix proportions defined per functional unit of 1 m^3^ of concrete.^84^ All material quantities, including cement, VA, water, fine aggregate, coarse aggregate, superplasticizer, and BA fiber, were directly taken from the mix design and expressed in kg/m^3^. No additional scaling factors were required because the mixtures were originally formulated on a per-cubic-meter basis.

For mixtures incorporating VA, cement was partially replaced on a mass basis while maintaining a constant total binder content. This approach ensures that environmental differences among mixtures are directly linked to material substitution rather than changes in total binder dosage. Fine and coarse aggregates were held constant across all mixtures, allowing variations in environmental impact to be attributed primarily to binder modification and BA fiber inclusion.

Since BA fiber was defined by volume fraction, its mass per functional unit was calculated using its material density. The mass of BA fiber in each mixture was determined as

1 $$m_{BA}=\rho_{BA}\times \left(\frac{V_{BA}}{100}\right)$$

where m_BA_ is the mass of BA fiber (kg/m^3^), ρ_BA_ is the density of BA fiber (kg/m^3^), and v_BA_ is the volume fraction of BA fibers expressed as a decimal (−).

The background inventory data were obtained from the GaBi database and selected to represent average industrial production conditions. The inventory included data sets associated with cement production, aggregate processing, water supply, chemical admixture production, basalt fiber production, road freight transportation, and marine freight transportation. Consistent background data sets were applied throughout the study to ensure methodological comparability among all investigated mixtures. Transportation-related inventory parameters, including transport distances, freight modes, and emission factors, were incorporated separately as described in Section 4.3.

All material production data sets were selected from the GaBi database to represent average industrial production conditions, while transportation processes were modeled separately using road freight and marine freight inventory data sets. By normalizing all inputs to 1 m^3^ and applying consistent background inventory data, the LCI framework ensures methodological consistency and comparability among all investigated mixtures.

### 4.3 Transportation Modeling and Assumptions

Transportation impacts were modeled by considering the actual supply chain routes for each constituent delivered to the batching plant in Van, Türkiye. All transport contributions were calculated on a ton·km basis and integrated into the LCA model using road freight and marine freight transportation data sets available in the GaBi database.

Cement was assumed to be transported by 30-ton road freight trucks over a distance of 970 km from the cement factory to the batching plant. Due to the relatively high cement content in the mixtures, this long-distance transport represents a significant contributor to transport-related environmental impacts.

VA was transported from Patnos to Van over an estimated distance of 150 km. Compared to cement, VA has a substantially shorter transport distance, which partially offsets the environmental burden associated with binder materials. Fine and coarse aggregates were assumed to be supplied from local quarries within the urban or peri-urban region of Van, with an average road transport distance of 40 km. Although aggregates constitute the largest mass fraction of concrete, their short transport distance limits their relative contribution to transportation impacts. The superplasticizer (SP) was procured from a local distributor in Van and delivered by road over an estimated distance of 10 km. Owing to its low dosage and short transport route, its contribution to the overall environmental balance remains negligible.

BA fiber was assumed to be imported from China. The transportation chain consisted of marine freight transport from China to Mersin Port, Türkiye, followed by domestic road freight transportation from Mersin to Van. The sea transport distance was set at 3477 nautical miles (6439.40 km), while the domestic road transport distance from Mersin Port to Van was assumed to be 970 km. This combined sea–road transport chain was modeled using marine freight and 30-ton road freight data sets available in the GaBi database.

For road transport, an emission factor of 62 g CO_2_ per ton·km was adopted as the base-case assumption. This value represents a representative average for heavy-duty freight vehicles operating under typical transport conditions.^85^ All transport-related impacts were calculated using transported mass,^86^ transportation distance,^87^ freight mode, and corresponding emission factors,^88^ ensuring methodological consistency across all investigated mixtures.

Given the imported nature of BA fibers, a brief sensitivity evaluation was also considered. Although BA fiber transportation involves relatively long transport distances, its contribution to total environmental impacts remains limited due to the low fiber dosage used in the mixtures (0.50–1.00% by volume). Therefore, moderate variations in transportation distance or freight assumptions are not expected to alter the overall environmental ranking of the investigated mixtures, which remains primarily governed by cement consumption and volcanic ash substitution.

### 4.4 Impact Assessment Methodology

The environmental impacts of the investigated mixtures were evaluated using a midpoint impact assessment approach implemented in GaBi. The selected impact categories were GWP, AP, and EP, as these indicators are widely used to assess the environmental performance of cement-based materials.

GWP quantifies greenhouse gas emissions and is expressed in kg CO_2_-equivalent per functional unit (kg CO_2_-eq/m^3^). This category primarily reflects CO_2_ emissions associated with cement production and transportation processes. AP represents emissions that contribute to atmospheric acid formation and is expressed in kg SO_2_-equivalent per m^3^ (kg SO_2_-eq/m^3^). EP measures the potential for nutrient enrichment in aquatic systems and is expressed in kg PO_4_^3 –^ -equivalent per m^3^ (kg PO_4_-eq/m^3^).

For each mixture, the total impact in a given category (X) was calculated as the sum of material production impacts and transportation impacts:

2 $$X_{Total}=\Sigma \left(m_{i}.X_{i}\right)+\Sigma \left(\frac{m_{i}}{1000}\right).d_{i}.X_{Transport}$$

where X_Total_ represents the total impact of the mixture per cubic meter (per m^3^), m_i_ is the mass of material *i*(kg/m^3^), X_i_ denotes the unit environmental impact of material *i* (impact/kg), d_i_ is the transport distance of material *i*(km), and X_transport_ is the transport emission factor (impact per ton·km).

For BA fiber, which involves both marine and road transport, the transport contribution was calculated as

3 $$X_{BA-transport}=\left(\frac{m_{BA}}{1000}.d_{sea}.X_{sea}\right)+\left(\frac{m_{BA}}{1000}.d_{road}.X_{road}\right)$$

Where X_BA_transport_ represents the transportation-related environmental impact of BA fiber per cubic meter of concrete, m_BA_ is the mass of BA fiber (kg/m^3^), d_sea_ is the sea transport distance (km), droad is the domestic road transport distance (km), Xsea represents the marine freight emission factor (impact per ton·km), and X_road_ denotes the road freight emission factor (impact per ton·km).

This calculation framework ensures that both material production and logistics-related contributions are consistently integrated for all mixtures. All impacts were normalized to the functional unit of 1 m^3^ of concrete to enable direct comparison between mixtures with different VA and BA fiber contents.

### 4.5 Environmental Results and Interpretation

The environmental performance of the mixtures was evaluated in terms of GWP, AP, and EP, and the results are presented in Figure 15. All impacts were calculated per functional unit (1 m^3^ of concrete), enabling a consistent comparison among mixtures with varying VA and BA fiber contents.

**Figure 15:**
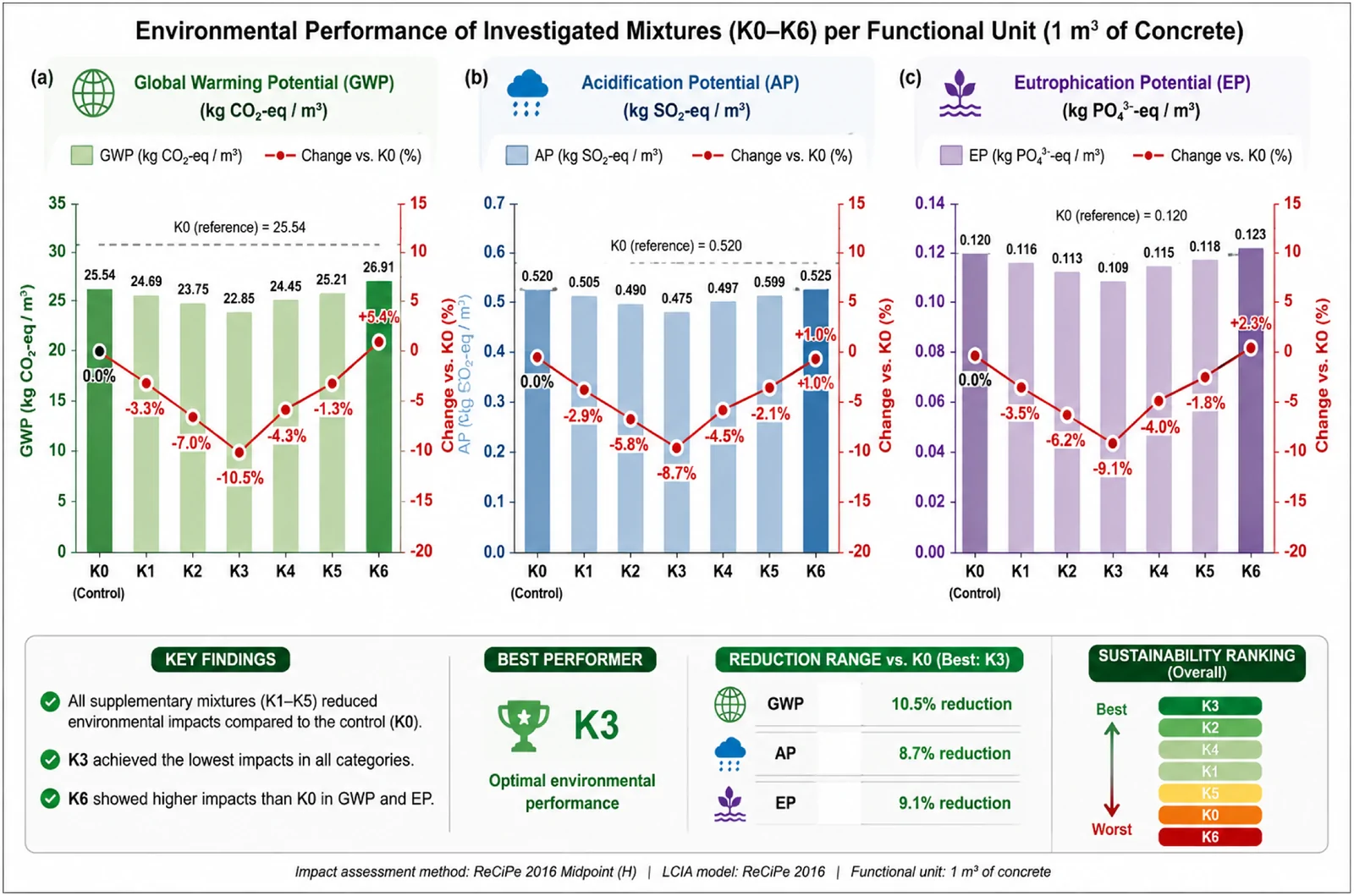
Comparative environmental impact indicators of the investigated mixtures (K0–K6) expressed per functional unit (1 m^3^ of concrete): (a) global warming potential (GWP), (b) acidification potential (AP), and (c) eutrophication potential (EP).

Figure 15(a) presents the global warming potential (GWP) results of mixtures K0–K6. The results demonstrate a gradual reduction in GWP values from K0 to K3 as the volcanic ash (VA) replacement ratio increases. This decrease is primarily attributable to a reduction in Portland cement consumption, the dominant source of embodied CO_2_ emissions in the mixtures, due to its energy-intensive production process and a long-distance transportation route (970 km).^89^ The partial replacement of cement with VA therefore contributed to a noticeable reduction in the environmental impact of the recycled aggregate concrete mixtures.

Among the VA-containing mixtures, K2 incorporating 10% VA exhibited the most balanced performance in terms of both environmental impact and mechanical behavior. At this replacement level, the reduction in cement consumption was achieved without significant deterioration in the compressive strength and durability of the mixtures. However, when basalt (BA) fibers were incorporated into the mixtures (K4–K6), the GWP values gradually increased as the BA fiber volume fraction increased. This increase can be attributed to the additional environmental burden associated with BA fiber production as well as the long-distance transportation process involving both sea and road transport routes. Consequently, although VA incorporation significantly improved the environmental sustainability of the mixtures by reducing cement-related emissions, excessive BA fiber incorporation partially offset these gains due to additional embodied carbon associated with fiber production and transportation.

A sensitivity consideration further indicated that the environmental contribution of BA fibers remained relatively limited despite the long transportation route from China to Türkiye and subsequent road transport to Van. This finding is primarily attributed to the low BA fiber dosage used in the mixtures (0.50–1.00% by volume). Therefore, moderate variations in BA fiber transportation distance or production-related assumptions are not expected to significantly alter the overall environmental ranking of the investigated mixtures. Instead, cement consumption and the level of volcanic ash substitution remained the dominant factors governing the environmental performance of the recycled aggregate concrete mixtures.

The AP results, shown in Figure 15(b), follow a trend similar to that of GWP. Since AP is strongly influenced by emissions from cement manufacturing and diesel-powered transport,^90^ mixtures with reduced cement content exhibit lower AP values. As BA fiber content increases, AP values rise slightly due to additional transport-related emissions and the embodied impacts of fiber production. Nevertheless, the magnitude of variation in AP is smaller than that in GWP, indicating that cement substitution remains the dominant influencing factor.

Figure 15(c) presents the EP results. The EP behavior also reflects the combined influence of binder modification and fiber logistics.^91^ Moderate VA replacement reduces EP values due to lower cement demand. In contrast, higher BA fiber volume fractions lead to incremental increases in EP, primarily related to transport emissions and upstream production processes. Similar to AP, the variation range in EP among mixtures is moderate, suggesting that binder composition plays a more decisive role than fiber addition within the investigated limits.

## 5 Conclusion

This study presented a comprehensive evaluation of recycled aggregate concrete (RAC) incorporating volcanic ash (VA) as a sustainable supplementary cementitious material and basalt (BA) fibers as a crack-control reinforcement strategy. Unlike conventional mixture optimization studies that primarily focus on isolated mechanical properties, the present research integrated durability performance, regression-based correlation analysis, SEM/ImageJ-supported microstructural characterization, and environmental life cycle assessment within a unified evaluation framework. The findings demonstrated that moderate VA incorporation significantly improved matrix compactness, pore refinement, and transport resistance, thereby reducing water absorption and chloride-ion permeability while enhancing compressive performance. The optimum behavior was observed at 10% VA replacement, where the combined influence of filler effects and pozzolanic reactions led to the development of a denser, more mechanically stable cementitious structure. However, excessive VA incorporation reduced matrix continuity due to dilution caused by insufficient cement hydration.

The incorporation of BA fibers revealed a more complex mechanical and durability response governed by fiber–matrix interaction and microstructural heterogeneity. Low BA fiber incorporation (0.25%) improved compressive strength through crack-bridging and stress redistribution mechanisms, whereas higher fiber contents increased localized void formation, interfacial discontinuities, and pore connectivity, negatively influencing both mechanical performance and transport-related durability properties. Regression analyses confirmed that durability-related parameters were strongly associated with compressive and flexural behavior, highlighting the critical role of pore connectivity and internal transport mechanisms in determining the overall structural performance of the mixtures. SEM observations and ImageJ-based void segmentation analysis further verified that VA incorporation reduced matrix porosity and improved interfacial compactness, whereas BA fibers altered local pore distribution through fiber-induced interfacial effects.

From a sustainability perspective, incorporating VA significantly reduced environmental burdens by decreasing cement consumption and associated CO_2_ emissions, whereas increasing BA fiber dosage increased environmental impacts due to energy-intensive production and transportation processes. These findings demonstrate that the sustainability and performance efficiency of RAC systems depend on achieving an optimal balance among the utilization of supplementary cementitious materials, fiber reinforcement efficiency, and microstructural stability. Overall, the present study advances current understanding of the coupled relationship between pore structure evolution, transport behavior, mechanical response, and environmental performance in VA- and BA-modified recycled aggregate concrete systems. Future studies should further investigate long-term durability performance under aggressive environmental conditions and explore AI-assisted mixture optimization and multiscale modeling approaches to improve the predictive design and sustainability efficiency of advanced recycled concrete composites. The observed performance trend suggests an optimal VA replacement threshold. While moderate VA contents enhanced matrix densification through filler and pozzolanic effects, excessive replacement levels reduced the availability of cement hydration products and weakened matrix continuity due to dilution effects. Therefore, the optimum performance observed at 10% VA reflects the balance between these competing mechanisms.

## Data Availability

Data supporting this study are available from the corresponding author upon reasonable request.

## References

[1] Ozcelikci E., Hu M., van der Meide M., Sahmaran M. (2025). Life cycle assessment
of SCM substitution in various CDW-based geopolymer concretes and
sensitivity analyses on allocation methods. Waste Manag..

[2] Lai M. H., Peng W. J., Xie Y. M., Ho J. C. M., Kitipornchai S., Ren F. M. (2026). Wet packing density-based
design for improving passing
ability and strength of recycled aggregate concrete. J. Build. Eng..

[3] Gao Y., Zhang S., Wang S., Jiang H., Sui H., Zhao L. (2026). Aggregate
particle size distribution impact on the microstructure
characteristics in the interface transition zone of recycled concrete. Constr. Build. Mater..

[4] Assaad J. J., Şahin H. G., Kaplan G., Mardani A. (2026). Alternatives to mitigate
the inferior performance of wet-mix shotcrete containing high recycled
fine aggregate replacement rates. Eur. J. Environ.
Civ. Eng..

[5] Zhang Z., Jiang Y., Zou Y., Zhou H., Yang J., Li Y., Xia J. R. (2026). Microscopic
damage mechanism of recycled aggregate
concrete under coupled freeze-thaw and physical sulfate attack in
semi-buried conditions. Constr. Build. Mater..

[6] Zhuofan G. U., Jiehui W. A. N. G. (2025). Experimental
study on crack surface characteristics
for aggregate interlock in natural and recycled aggregate concrete. J. Build. Eng..

[7] Xu Z., Li S. (2025). Evaluation of sulfate
corrosion-dry–wet cycle resistance in
concrete incorporating heterogeneous recycled brick/stone coarse aggregates. J. Build. Eng..

[8] Xiao J., Yu C., Wang B., Liu H., Poon C. S., de Brito J., Shah S. P. (2026). Recycled aggregate concrete design, application and
challenges. Nat. Rev. Clean Technol..

[9] Ramalingam M., Sivamani J., Narayanan K. (2023). Performance
studies on recycled aggregate
concrete with treated recycled aggregates. Waste
Disposal Sustainable Energy.

[10] Singh R. P., Vanapalli K. R., Jadda K., Mohanty B. (2024). Durability assessment
of fly ash, GGBS, and silica fume based geopolymer concrete with recycled
aggregates against acid and sulfate attack. J. Build. Eng..

[11] Kumar A., Jail Singh G., Chauhan B. L., Kumar R. (2024). Strength and durability
performance of recycled aggregate structural concrete with silica
fume, furnace slag, and M-Fine. J. Mater. Civ.
Eng..

[12] Al-Naghi A. A. A., Ali T., Inam I., Qureshi M. Z., Kahla N. B., Ghazouani N. (2025). An innovative
approach to enhancing the strength and
durability of recycled aggregate concrete through fly ash-silica fume
coating and rice husk ash supplementation. Sci.
Rep..

[13] Hamada H. M., Abed F., Beddu S., Humada A. M., Majdi A. (2023). Effect of
Volcanic Ash and Natural Pozzolana on mechanical properties of sustainable
cement concrete: A comprehensive review. Case
Stud. Constr. Mater..

[14] Ahmad J., Althoey F., Abuhussain M. A., Deifalla A. F., Özkılıç Y. O., Rahmawati C. (2023). Durability and microstructure analysis of concrete
made with volcanic ash: A review (Part II). Sci. Eng. Compos. Mater..

[15] Guler S., Akbulut Z. F. (2023). Workability & mechanical properties of the single
and hybrid basalt fiber reinforced volcanic ash-based cement mortars
after freeze–thaw cycles. Structures.

[16] Anwar
Hossain K. M., Ahmed S. (2011). Lightweight concrete incorporating
volcanic ash-based blended cement and pumice aggregate. J. Mater. Civ. Eng..

[17] Chakkamalayath J., Joseph A., Al-Baghli H., Hamadah O., Dashti D., Abdulmalek N. (2020). Performance
evaluation of self-compacting concrete
containing volcanic ash and recycled coarse aggregates. Asian J. Civil Eng..

[18] Letelier V., Ortega J. M., Tarela E., Muñoz P., Henríquez-Jara B. I., Moriconi G. (2018). Mechanical
performance
of eco-friendly concretes with volcanic powder and recycled concrete
aggregates. Sustainability.

[19] Aslani F., Hou L., Nejadi S., Sun J., Abbasi S. (2019). Experimental analysis
of fiber-reinforced recycled aggregate self-compacting concrete using
waste recycled concrete aggregates, polypropylene, and steel fibers. Structural Concrete.

[20] Caggiano A., Gambarelli S., Martinelli E., Nisticò N., Pepe M. (2016). Experimental characterization
of the post-cracking response in hybrid
steel/polypropylene fiber-reinforced concrete. Constr. Build. Mater..

[21] Zhang T., Wang K., Lin B., Yao Y. (2024). The enhancement mechanism
of modified basalt fiber on the performance of geopolymer concrete. Constr. Build. Mater..

[22] Khan M., Cao M., Xie C., Ali M. (2022). Effectiveness of hybrid steel-basalt
fiber reinforced concrete under compression. Case Studies in Construction
Materials. Constr. Build. Mater..

[23] Zheng Y., Zhuo J., Zhang Y., Zhang P. (2022). Mechanical
properties
and microstructure of nano-SiO2 and basalt-fiber-reinforced recycled
aggregate concrete. Nanotechnol. Rev..

[24] Wang Y., Kang A. H., Wu Z. G., Xiao P., Gong Y. F., Sun H. F. (2023). Investigation of the basalt fiber type and content
on performances of cement mortar and concrete. Constr. Build. Mater..

[25] Patrisia Y., Tran N. P., Gunasekara C., Law D. W., Ngo T. D., Setunge S. (2026). Potential SCMs for low-carbon concrete in Australia:
Cradle-to-gate LCA and cost perspectives. Resour.
Conserv. Recycl..

[26] Sanjay Shanbhag, S. ; Kumar Dixit, M. Evaluating the GWP of Hempcrete vs. Concrete in Non-Load-Bearing Applications: A Case Study of a Single-Family Home AEI 2025: delivering Future Ready Buildings—An Integrated Approach AEI 2026 348–356

[27] Ramezani A., Pepera R., Shafei B. (2026). Performance
and life cycle assessment
of fiber-reinforced concrete mixtures with polypropylene, polyvinyl
alcohol, and alkali-resistant glass fibers. Cleaner Mater..

[28] Alhashash M., Alariyan A., Youns A. M., Ghreivati F., Habib A., Habib M. (2026). Recent Efforts on the Compressive
and Tensile Strength Behavior of Thermoplastic-Based Recycled Aggregate
Concrete toward Sustainability in Construction Materials. Struct. Durab. Health Monit..

[29] Sun Q., Wu Y. F. (2026). Influence of recycled coarse aggregate quality on carbonation
behavior
of compression-cast fully recycled aggregate concrete. Constr. Build. Mater..

[30] ASTM International ASTM C618–19 “Standard specification for coal fly ash and raw or calcined natural pozzolan for use in concrete”; ASTM International, West Conshohocken, PA, USA, 2019.

[31] ASTM International. (2013). ASTM C642: standard Test Method for Density, Absorption, and Voids in Hardened Concrete. ASTM International, West Conshohocken, PA.

[32] Turkish Standards Institution (TSE). (2019). TS EN 12390–3: testing hardened concrete – Part 3: compressive strength of test specimens. TSE, Ankara.

[33] Turkish Standards Institution (TSE). (2019). TS EN 12390–5: testing hardened concrete – Part 5: flexural strength of test specimens. TSE, Ankara.

[34] ASTM International. (2019). ASTM C1202: standard Test Method for Electrical Indication of Concrete’s Ability to Resist Chloride Ion Penetration. ASTM International: West Conshohocken, PA.

[35] Ettahiri Y., Vico Lujano R., Bouna L., Benlhachemi A., Cáceres-Alvarado J. M., Eliche-Quesada D., Pérez-Villarejo L. (2026). Valorization of Volcanic
Ash and
Stainless Steel Slag as Partial Replacements of Metakaolin in Geopolymer
Binders. Materials.

[36] Bonen D., Shah S. P. (2005). Fresh and hardened
properties of self-consolidating
concrete. Prog. Struct. Eng. Mater..

[37] Chu S. H. (2019). Effect
of paste volume on fresh and hardened properties of concrete. Constr. Build. Mater..

[38] High C., Seliem H. M., El-Safty A., Rizkalla S. H. (2015). Use of basalt fibers
for concrete structures. Constr. Build. Mater..

[39] Wu J., Pang Q., Lv Y., Zhang J., Gao S. (2022). Research on
the mechanical and physical properties of basalt fiber-reinforced
pervious concrete. Materials.

[40] Falliano D., De Domenico D., Ricciardi G., Gugliandolo E. (2019). Compressive
and flexural strength of fiber-reinforced foamed concrete: Effect
of fiber content, curing conditions and dry density. Constr. Build. Mater..

[41] Cai G., Noguchi T., Degée H., Zhao J., Kitagaki R. (2016). Volcano-related
materials in concretes: a comprehensive review. Environ. Sci. Pollut. Res..

[42] Kumar B. N., Neelamegam P., Sai A. P. D., Berhe M. A. (2026). Investigation of
carbonation-induced microstructural changes in low-cement concrete
using sustainable binders: GGBS and calcium carbonate. Sci. Rep..

[43] Játiva A., Ruales E., Etxeberria M. (2021). Volcanic ash
as a sustainable binder
material: An extensive review. Materials.

[44] Naseer A., Jabbar A., Khan A. N., Ali Q., Hussain Z., Mirza J. (2008). Performance of Pakistani volcanic
ashes in mortars and concrete. Can. J. Civ.
Eng..

[45] Divyah N., Thenmozhi R., Neelamegam M., Prakash R. (2021). Characterization and
behavior of basalt fiber-reinforced lightweight concrete. Structural Concrete.

[46] Liu Q., Song P., Li L., Wang Y., Wang X., Fang J. (2022). The effect of basalt
fiber addition on cement concrete: A review
focused on basalt fiber shotcrete. Front. Mater..

[47] Mousavinezhad S., Nozari S., Newtson C. M. (2025). Classifying Concrete
Permeability
Using Rapid Chloride Permeability and Surface Resistivity Tests: Benefits,
Limitations, and Predictive Models—A State-of-the-Art Review. Buildings.

[48] Ibrahim M., Bahraq A. A., Salami B. A., Alhems L. M., Hussaini S. R., Nasir M., Adewumi A. A. (2025). Pore Structure
Characterization and
Environmental Assessment of Ground Volcanic Pumice-Based Alkali-Activated
Concrete. Int. J. Concr Struct Mater..

[49] Kasaniya M., Thomas M. D., Moffatt E. G. (2021). Pozzolanic
reactivity of natural
pozzolans, ground glasses and coal bottom ashes and implication of
their incorporation on the chloride permeability of concrete. Cem. Concr. Res..

[50] Aguirre-Guerrero A. M., Robayo-Salazar R. A., de Gutiérrez R.
M. (2021). Corrosion resistance
of alkali-activated binary reinforced concrete based on natural volcanic
pozzolan exposed to chlorides. J. Build. Eng..

[51] Guo Y., Hu X., Lv J. (2019). Experimental study on the resistance
of basalt fibre-reinforced
concrete to chloride penetration. Constr. Build.
Mater..

[52] Zhang L., Li L., Li F., Huang Z. (2025). Study on Rapid Chloride Ion Migration,
Frost Resistance and Microstructure Evolution of Basalt Fiber Reinforced
Cement-Based Composites. J. Railw. Sci. Technol..

[53] Ding X., Liang X., Zhang Y., Fang Y., Zhou J., Kang T. (2020). Capillary water absorption
and micro pore connectivity of concrete
with fractal analysis. Crystals.

[54] Delmelle P., Villiéras F., Pelletier M. (2005). Surface area, porosity and water
adsorption properties of fine volcanic ash particles. Bull. Volcanol.

[55] Karolina R., Putra A. L. A. (2018). The effect of
steel slag as a coarse aggregate and
Sinabung volcanic ash a filler on high strength concrete. IOP Conf. Ser.: Mater. Sci. Eng..

[56] Bogas J. A., Gomes T. (2015). Mechanical and durability
behaviour of structural lightweight concrete
produced with volcanic scoria. Arabian J. Sci.
Eng..

[57] Abdulazeez A. S., Idi M. A., Justin T., Hamza B. (2020). Strength performance
of concrete produced with volcanic ash as partial replacement of cement. Int. J. Eng. Res. Technol..

[58] Yang L., Xie H., Fang S., Huang C., Yang A., Chao Y. J. (2021). Experimental
study on mechanical properties and damage mechanism of basalt fiber
reinforced concrete under uniaxial compression. Structures.

[59] John V. J., Dharmar B. (2021). Influence of basalt fibers on the mechanical behavior
of concrete—A review. Structural Concrete.

[60] Liu B., Shi J., Zhou F., Shen S., Ding Y., Qin J. (2020). Effects of
steam curing regimes on the capillary water absorption of concrete:
Prediction using multivariable regression models. Constr. Build. Mater..

[61] Awolusi T. F., Oke L. O., Akinkurolere O. O., Ubani D. P., Bamisaye R. T., Aluko O. G. (2021). The application of response surface methodology in
understanding the compressive strength and water absorption capacity
of sandcrete blocks. Silicon.

[62] Dhinakaran G., Thilgavathi S., Venkataramana J. (2012). Compressive strength and chloride
resistance of metakaolin concrete. KSCE J. Civ.
Eng..

[63] Presa L., Rosado S., Peña C., Martín D. A., Costafreda J. L., Astudillo B., Parra J. L. (2023). Volcanic ash from
the island of La Palma, Spain: An experimental study to establish
their properties as pozzolans. Processes.

[65] Zeyad A. M., Khan A. H., Tayeh B. A. (2020). Durability
and strength characteristics
of high-strength concrete incorporated with volcanic pumice powder
and polypropylene fibers. J. Mater. Res. Technol..

[66] Alnahhal W., Aljidda O. (2018). Flexural behavior of basalt fiber reinforced concrete
beams with recycled concrete coarse aggregates. Constr. Build. Mater..

[67] Shafiq N., Ayub T., Khan S. U. (2016). Investigating
the performance of
PVA and basalt fibre reinforced beams subjected to flexural action. Compos. Struct..

[68] Khoury E., Ambrós W., Cazacliu B., Sampaio C. H., Remond S. (2018). Heterogeneity
of recycled concrete aggregates, an intrinsic variability. Constr. Build. Mater..

[69] Bonifazi, G. ; Capobianco, G. ; Serranti, S. ; Eggimann, M. ; Wagner, E. ; Di Maio, F. ; Lotfi, S. The ITZ in concrete with natural and recycled aggregates: Study of microstructures based on image and SEM analysis Proceedings of the 15th Euroseminar on Microscopy Applied to Building Materials (15th EMABM) Delft University of Technology 2015 299–308

[70] Ren H., Qian Z., Lin B., Huang Q., Crispino M., Ketabdari M. (2022). Effect of
recycled concrete aggregate features on adhesion
properties of asphalt mortar-aggregate interface. Constr. Build. Mater..

[71] Kim Y., Hanif A., Usman M., Park W. (2019). Influence of bonded
mortar of recycled concrete aggregates on interfacial characteristics–Porosity
assessment based on pore segmentation from backscattered electron
image analysis. Constr. Build. Mater..

[72] Miao J., Jiang H., Wang S., Wang S., Huo J., Lin J., Gao Y. (2025). Influence
of graphene oxide on the recycled aggregate
concrete reinforcement under different particle size distribution. Constr. Build. Mater..

[73] Feng C., Wang J., Chen M., Zhao X., Zhang W., Zhu J., Du M. (2025). Study on the modification effect of recycled fine aggregates
treated by synergistic alkali activation using volcanic ash and cement
powder wrapping. J. Mater. Res. Technol..

[74] Santillán L. R., Zaccardi Y. A. V., Zega C. J., Méndez E. M. (2023). Macroscopic
behavior and microstructural analysis of recycled aggregate mortar
bars exposed to external sulfate attack. Cement
Concrete Composites.

[75] Hou Y., Zhang X., Liu J., He C., Ji X. (2024). Interface
adhesion enhancement and multi-scale evaluation of recycled concrete
aggregate (RCA) and asphalt. Constr. Build.
Mater..

[76] Tahwia A. M., Abdellatief M., Salah A., Youssf O. (2025). Valorization of recycled
concrete powder, clay brick powder, and volcanic pumice powder in
sustainable geopolymer concrete. Sci. Rep..

[77] Hong L., Chen Y. D., Li T. D., Gao P., Sun L. Z. (2020). Microstructure
and bonding behavior of fiber-mortar interface in fiber-reinforced
concrete. Constr. Build. Mater..

[78] Wang D., Wu Y., Xu Z., Xu N., Li C., Tian X., Shi F., Wang H. (2025). Effect of
Basalt Fibers on the Performance of CO2-Cured
Recycled Aggregate Concrete Composite Slab–Column Assemblies
with Bolted Connections Under NaCl Erosion. Coatings.

[79] Liu J., Liu Q., Pu C., Zhu C., Li Y., Zhou J., Xu Y. (2025). Synergistic effects
and mechanisms of basalt fibers and polycarboxylate
superplasticizer on cement–fly ash stabilized aeolian sand
and crushed stones. PLoS One.

[80] Serres N., Braymand S., Feugeas F. (2016). Environmental
evaluation of concrete
made from recycled concrete aggregate implementing life cycle assessment. J. Build. Eng..

[81] Bennett B., Visintin P., Xie T. (2022). Global warming potential
of recycled
aggregate concrete with supplementary cementitious materials. J. Build. Eng..

[82] Park W. J., Kim T., Roh S., Kim R. (2019). Analysis of life cycle environmental
impact of recycled aggregate. Appl. Sci..

[83] Colangelo F., Navarro T. G., Farina I., Petrillo A. (2020). Comparative LCA of
concrete with recycled aggregates: A circular economy mindset in Europe. Int. J. Life Cycle Assess.

[84] Yazdanbakhsh A., Bank L. C., Baez T., Wernick I. (2018). Comparative LCA of
concrete with natural and recycled coarse aggregate in the New York
City area. Int. J. Life Cycle Assess.

[85] Visintin P., Dadd L., Alam M. U., Xie T., Bennett B. (2022). Flexural performance
and life-cycle assessment of multi-generation recycled aggregate concrete
beams. J. Clean. Prod..

[86] McIntyre J., Spatari S., MacLean H. L. (2009). Energy and greenhouse gas emissions
trade-offs of recycled concrete aggregate use in nonstructural concrete:
A North American case study. J. Infrastruct.
Syst..

[87] Pradhan S., Tiwari B. R., Kumar S., Barai S. V. (2019). Comparative LCA
of recycled and natural aggregate concrete using Particle Packing
Method and conventional method of design mix. J. Clean. Prod..

[88] Sabău M., Bompa D. V., Silva L. F. (2021). Comparative carbon
emission assessments
of recycled and natural aggregate concrete: Environmental influence
of cement content. Geosci. Front..

[89] Marinković S., Radonjanin V., Malešev M., Ignjatović I. (2010). Comparative
environmental assessment of natural and recycled aggregate concrete. Waste Manag..

[90] Namdari M. (2025). Comparative
LCA of diesel and grid electricity for agricultural irrigation in
Iran: why diesel outperforms in fossil-reliant grids. PLoS One.

[91] Sekhri K., Sudan N., Gopakumar N., Biligiri K. P. (2026). Balancing Performance
and Environmental Impacts of Crumb Rubber-Modified Asphalt Binders
using a Multi-Objective Lifecycle Optimization Process. Resour. Conserv. Recycl. Adv..

